# Microscale insights into pneumococcal antibiotic mutant selection windows

**DOI:** 10.1038/ncomms9773

**Published:** 2015-10-30

**Authors:** Robin A. Sorg, Jan-Willem Veening

**Affiliations:** 1 Molecular Genetics Group, Groningen Biomolecular Sciences and Biotechnology Institute, Centre for Synthetic Biology, University of Groningen, Nijenborgh 7, Groningen 9747 AG, The Netherlands

## Abstract

The human pathogen *Streptococcus pneumoniae* shows alarming rates of antibiotic resistance emergence. The basic requirements for *de novo* resistance emergence are poorly understood in the pneumococcus. Here we systematically analyse the impact of antibiotics on *S. pneumoniae* at concentrations that inhibit wild type cells, that is, within the mutant selection window. We identify discrete growth-inhibition profiles for bacteriostatic and bactericidal compounds, providing a predictive framework for distinction between the two classifications. Cells treated with bacteriostatic agents show continued gene expression activity, and real-time mutation assays link this activity to the development of genotypic resistance. Time-lapse microscopy reveals that antibiotic-susceptible pneumococci display remarkable growth and death bistability patterns in response to many antibiotics. We furthermore capture the rise of subpopulations with decreased susceptibility towards cell wall synthesis inhibitors (heteroresisters). We show that this phenomenon is epigenetically inherited, and that heteroresistance potentiates the accumulation of genotypic resistance.

## Abstract (web summary)

The emergence of antibiotic resistance in bacteria is driven by inhibitory but non-lethal antibiotic concentrations. Here, Sorg and Veening study the effects of different antibiotics on the pneumococcus, with a focus on inhibition dynamics, metabolic activity and processes at the single-cell level.

## Graphical abstract

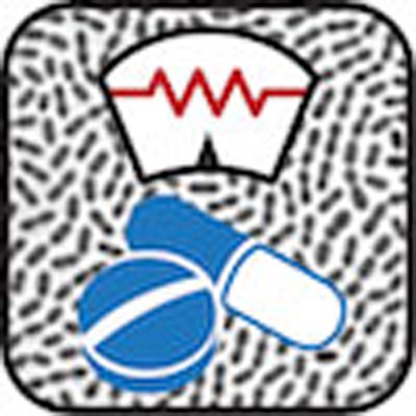

A report of the World Health Organization from 2014 described a dramatic worldwide increase in antibiotic resistance among human pathogens^1^. This issue is not only caused by a decline in discoveries of new antibiotics but also by drug application practices that lack insight into bacterial resistance development^2^. *Streptococcus pneumoniae* (the pneumococcus) is a prominent example of a human pathogen that was shown to quickly evolve and become refractory to antibiotic therapy^3^. The pneumococcus is a worldwide health threat that is responsible for more than one million deaths per year^4^. Pneumococci are commonly found in a commensal state in the nasopharynx of 50% of healthy children and 10% of healthy adults^5^,^6^. These high carriage frequencies (and therefore also regular drug exposures), in combination with the characteristic of pneumococci to develop natural competence (allowing for DNA uptake and chromosomal integration), are believed to be major driving forces for rapid resistance acquisition^7^.

Standard treatments of invasive pneumococcal infections, such as meningitis, sepsis or pneumonia, involve the administration of antibiotics. However, the impact of antibiotics on the pneumococcal cell physiology is poorly characterized. Conventional concepts for drug impact are based on population assays and growth evaluation after overnight incubation, specifically the formation of visible colonies or medium turbidity. This approach is straightforward but provides no information about doubling times, and only limited information about cell-to-cell variability. Decreased bacterial growth rates, however, are important for the enrichment of cells that are less susceptible towards antibiotics, and mutagenesis towards genotypic resistance requires viable cells. Antibiotic resistance development is therefore driven by inhibitory but non-lethal drug concentrations within the mutant selection window (MSW)^2^,^8^. During antibiotic therapy, the non-uniform dispersal of drug molecules inside the human body can result in local antibiotic concentration ranges that promote resistance development^9^. Antibiotic treatment of bacterial cultures has, furthermore, been shown to give rise to subpopulations with decreased drug susceptibility, a factor that might influence resistance evolution^10^,^11^. One mechanism is bacterial persistence, in which populations bifurcate into many growing and few non-growing cells^12^. Another phenomenon is found in heteroresistance towards cell wall synthesis inhibitors, in which only a fraction of the cell population emerges as colony-forming units (CFUs) at drug concentrations that inhibit the overall population^13^,^14^. For both examples, it remains unclear whether antibiotic treatment triggers the observed cell-to-cell variability, or whether pre-existing heterogeneity within bacterial populations becomes exposed^15^.

While some factors for resistance development are specific for individual antibiotics and their mode of action, others are more general, such as cell survival. Recently, the increased production of reactive oxygen species via the electron transport chain has been hypothesized to contribute to cell death for diverse bactericidal antibiotics^16^,^17^,^18^,^19^,^20^. This mechanism cannot take place in the proposed way in *S. pneumoniae* because genes encoding a complete electron transport chain are missing^21^,^22^. Furthermore, pneumococci do not exhibit a classical SOS response. Instead, they activate the developmental process of competence when inhibited by certain antibiotics^23^,^24^. Thus, differences in genetic background demonstrate that various species-specific reaction patterns to antibiotic stress exist that require case-by-case investigation.

Here we examine antibiotic treatment of *S. pneumoniae* in a systematic manner, both at the population level as well as at the single-cell level. We assay the impact of drug concentrations within the MSW, with a specific focus on inhibition dynamics, cellular metabolic activity and cell-to-cell variability. Mutation assays are carried out that highlight important prerequisites for the formation of resistant cell populations. Our results complement the picture of how the physiology of pneumococcal cells is affected by drug treatment, and indicate mechanisms that might be crucial for the understanding of resistance evolution and for supporting the development of novel antibiotic chemotherapy.

## Results

### Growth inhibition by bacteriostatic and bactericidal agents

Clinical isolates of *S. pneumoniae* are often resistant to many antibiotics that were frequently used for therapy. In the attempt to understand resistance evolution in a bottom-up approach, and to identify the key determinants for this development, we used the well-characterized antibiotic-susceptible strain D39 (derived from an early clinical isolate)^25^. Experiments were carried out in the derivative strain D-PEP22, which contains a genomic integration for constitutive luciferase expression, and luminescence served as a proxy for gene expression activity^26^. Twelve antibiotics, spanning most functional categories, were tested for their impact on the pneumococcus (Table 1). To test bulk growth behaviour, dose–response assays with concentration series of each antibiotic compound were carried out in liquid culture (see Methods).

**Figure 1:**
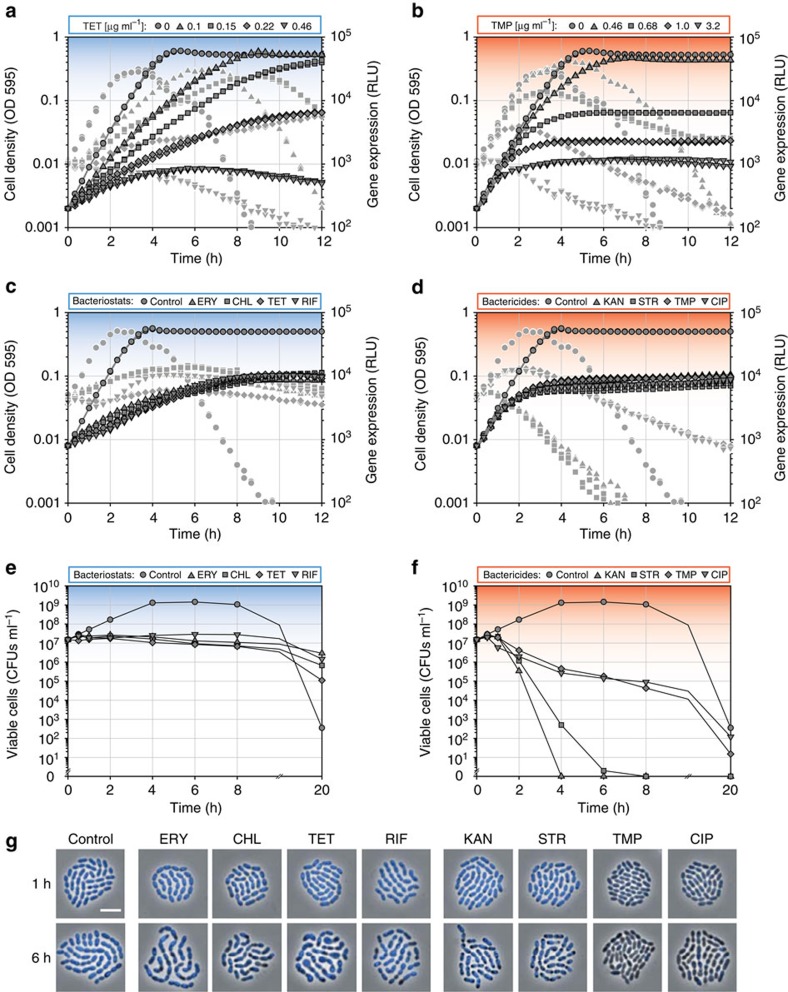
Impact of bacteriostatic and bactericidal antibiotics at F10 concentrations. (**a**,**b**) Plate reader assays measuring cell density (closed symbols, OD595) and gene expression (symbols without outline, RLU, relative luminescence unit, a.u.) of *S. pneumoniae* D-PEP22 (that constitutively expresses luciferase) growing in the presence of concentration series of a typical bacteriostatic (tetracycline, **a**) and a typical bactericidal (trimethoprim, **b**) antibiotic; experimental duplicates are shown and all experiments were replicated at least three times. (**c**,**d**) Comparison of cell density and gene expression kinetics during treatment with bacteriostatic (**c**) and bactericidal (**d**) antibiotics at F10 concentrations (allowing for OD increase in treated cultures of a factor 10 within 10 h of cultivation). CHL, chloramphenicol; CIP, ciprofloxacin; ERY, erythromycin; KAN, kanamycin; RIF, rifampicin; STR, streptomycin; TET, tetracycline; TMP, trimethoprim. (**e**,**f**) Development of the count of viable cells (CFUs ml^−1^) during F10 treatment; average values of duplicates are shown. (**g**) Overlay of phase contrast and fluorescence microscopy of Hoechst DNA-stained D-PEP22 taken from 1 h (upper panel) and 6 h (lower panel) of F10 treatment. Scale bar, 4 μm.

Strikingly, two distinct growth-inhibition profiles emerged. For antibiotics reported to be bacteriostatic (bacteriostats) in *S. pneumoniae* (with tetracycline as example in Fig. 1a; for complete list see Supplementary Fig. 1), the following relationship was found: the higher the applied concentration, the slower the bacterial growth is immediately from the starting point of drug exposure. For antibiotics previously described as bactericidal (bactericides) in the pneumococcus (with trimethoprim as example in Fig. 1b; for complete list see Supplementary Fig. 1), all applied drug concentrations allowed for an initial growth without noticeable inhibition, and the higher the applied concentration, the shorter the period of unimpaired growth. For both antibiotic classifications, gene expression profiles were in good agreement with their corresponding cell density profiles and confirmed the existence of two distinct inhibition patterns (Fig. 1a,b). Note that luminescence from stationary phase cultures, and thus from metabolically less active cells, decreased quickly because of the ATP dependence and short half-life of luciferase^27^.

To compare the impact of individual antibiotics on pneumococcal cell physiology in more detail, we first established a set of standard concentrations. The commonly used standard of minimal inhibitory concentration (MIC) is defined as the lower boundary of MSWs. MICs describe the antibiotic concentration required to visibly inhibit bacterial growth after overnight incubation^28^. However, the lack of growth at a macroscopic scale is not conclusive for bacterial growth behaviour at the microscopic scale; limited growth, such as four cell doublings, results in outcomes that cannot be detected by eye, such as medium turbidity increases of factor 16 (not detectable when assuming low inoculation densities) or microcolonies containing 16 cells. We therefore propose an antibiotic concentration standard independent of MIC values (yet above MICs) that limits optical density (OD) increase of treated cultures to a factor 10 within 10 h of cultivation (F10 concentrations; Table 1). While OD increase is not necessarily a good proxy for increases in CFUs (cells may change in size, composition or viability), it is proportional to cellular anabolic activity and thus indicative for nutrient consumption. Nutrient consumption rates are relevant for the enrichment of mutants in natural environments because MSWs describe conditions where resistant cells outcompete susceptible cells for common limited resources. Exposure to F10 concentrations in growth experiments with series of inoculation densities resulted in different final OD values; however, OD fold-increases between assays were similar, which emphasizes that logarithmic scales are crucial for visualizing bacterial growth phenomena (Supplementary Fig. 2).

### Pathway-specific antibiotic impact

To compare the response of *S. pneumoniae* to different antibiotics in a systematic manner, we examined exposure to F10 concentrations by monitoring cell growth and gene expression activity (Fig. 1c,d). Interestingly, cells that were growth-arrested by exposure to bacteriostatic drugs showed increased metabolic activity compared with cells of untreated cultures that growth-arrested upon reaching stationary phase (Fig. 1c). Cells treated with bactericidal drugs acting on DNA synthesis also displayed prolonged gene expression activity, whereas aminoglycoside antibiotics that inhibit translation caused a rapid gene expression shutdown (Fig. 1d). Plating assays revealed that cells of untreated cultures showed a substantial loss of viability after 20 h, presumably because of autolysis in the stationary phase (Fig. 1e)^29^. Remarkably, in the presence of bacteriostatic antibiotics, more cells remained viable after 20 h compared with untreated control cultures, indicating that bacteriostatic antibiotics at F10 concentrations protect from autolysis (Fig. 1e). Survival from treatment with bactericidal antibiotics depended on the targeted pathway; after 8 h, the number of viable cells dropped by more than two orders of magnitude for DNA synthesis inhibitors, while aminoglycosides caused the death of the entire population (Fig. 1f).

Bacteria that suffered from antibiotic treatment are known to display persisting inhibition even after drug removal, a phenomenon called the post-antibiotic effect^30^. To evaluate how *S. pneumoniae* recovers from antibiotic stress, we re-cultured cells in an antibiotic-free medium after defined periods of F10 exposure (Supplementary Fig. 3). In general, with increasing windows of exposure to bacteriostatic antibiotics, cells required more time to recover (longer lag phases). Time-lapse microscopy on cells coming from 1, 2 and 4 h of drug exposure confirmed this trend and showed that individual cells displayed lag heterogeneities. However, and in contrast to cells exposed to bactericidal antibiotics, all observed cells eventually grew out to form microcolonies. In cultures recovering from bactericidal drug treatment, population-wide gene expression shutdown continued for some amount of time, which indicates a large fraction of irreparably damaged cells that die off (Supplementary Fig. 3). For instance, after 4 h of ciprofloxacin treatment, luminescence continued to decrease for three more hours, and growth could not be observed before 5 h after drug removal (Supplementary Fig. 3).

These results prompted us to conduct phase contrast and fluorescence microscopy with Hoechst DNA staining to test whether prolonged drug exposure drives cells towards a progressively perturbed homeostasis (Fig. 1g). Inhibition of translation, for example, might impair some cell functions, such as membrane synthesis, to a different extent than others, such as DNA synthesis, and thus leads to alterations in cellular composition. Indeed, cells taken from one hour of F10 bacteriostatic drug exposure showed changes in nucleoid-staining intensity and distribution, and after 6 h perturbed cell morphologies emerged. Treatment with bactericidal drugs at F10 concentrations revealed that already after one hour of exposure, when cells still grew indistinguishably from the control assay, aminoglycoside-treated cells showed dark bodies at the cell poles indicative for protein aggregation^31^, and cells treated with DNA synthesis inhibitors showed greatly reduced fluorescence from DNA staining. These characteristics were even more pronounced after 6 h of drug exposure (Fig. 1g). The described experimental approach proved to be a straightforward strategy for monitoring pathway-specific cell damage in *S. pneumoniae*.

### Single-cell analysis uncovers growth heterogeneity

**Figure 2:**
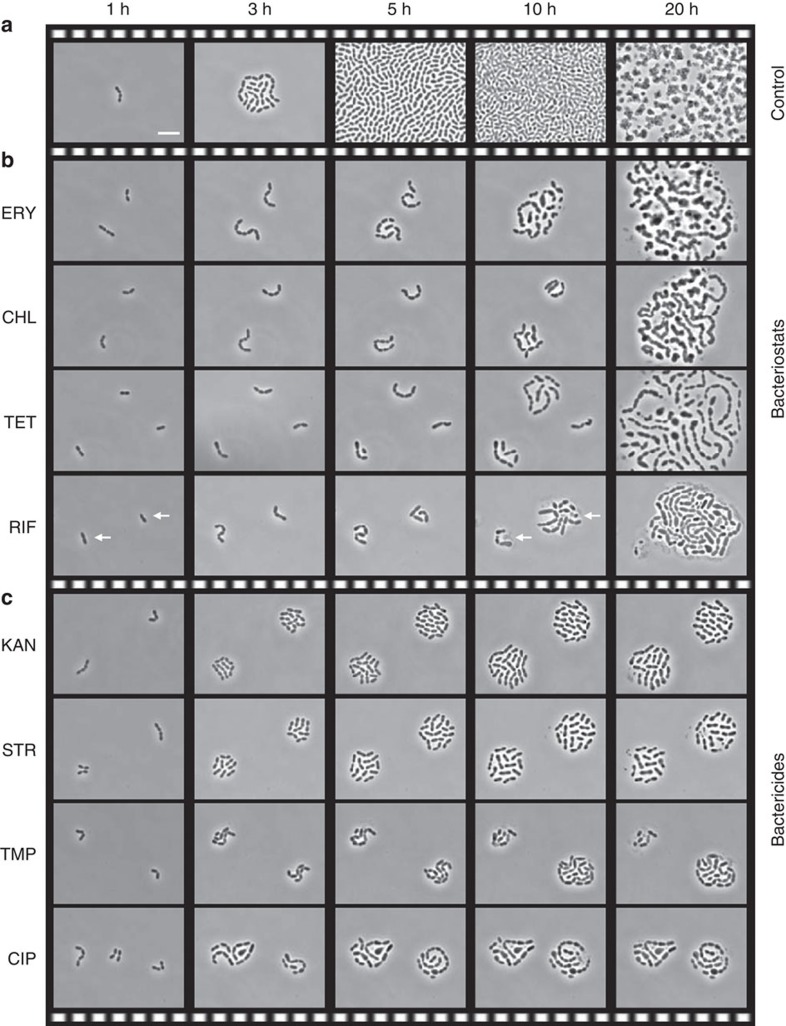
Time-lapse microscopy in the presence of F10 concentrations. (**a**) Still images (phase contrast microscopy) after 1, 3, 5, 10 and 20 h of a time-lapse experiment of *S. pneumoniae* D-PEP22 cells growing out to form microcolonies (and subsequent autolysis) on a semisolid surface without antibiotics. (**b**) Tracking of D-PEP22 cells treated with bacteriostatic antibiotics at the F10 level. Note the large amount of cell-to-cell variability, such as in case of rifampicin, where two cells develop in opposing directions (marked with arrows), with the progeny of one lysing while the other one grows out successfully. (**c**) Tracking of D-PEP22 cells treated with bactericidal antibiotics at the F10 level. CHL, chloramphenicol; CIP, ciprofloxacin; ERY, erythromycin; KAN, kanamycin; RIF, rifampicin; STR, streptomycin; TET, tetracycline; TMP, trimethoprim. Scale bar, 5 μm.

To follow the impact of F10 concentrations at the single-cell level, and to relate data of OD measurements to processes at a microscale, we performed time-lapse microscopy (Fig. 2 and Supplementary Movies 1 and 2). In general, the observation of distinct growth-inhibition profiles for bacteriostatic and bactericidal antibiotics in bulk measurements were confirmed. Bacteriostatic antibiotics gave rise to slowed but continuous growth, while bactericidal antibiotics did not affect cell doublings instantaneously but arrested growth with a delay (Fig. 2). Autolysis of *S. pneumoniae* during the stationary phase was confirmed and could be observed within 20 h of incubation (Fig. 2a). Antibiotic-treated cells on the other hand did not display synchronous lysis at this time and appeared mostly intact (Fig. 2b,c).

Pneumococci that were exposed to antibiotics with bacteriostatic activity showed high levels of cell-to-cell variability, both concerning growth rate at the single-cell level and perturbation of cell morphology (Fig. 2b and Supplementary Movie 1). For each individual bacteriostatic compound, a typical pattern of cell shapes and microcolony formation emerged. Exposure to antibiotics with bactericidal activity, in contrast, demonstrated surprisingly little cell-to-cell variability (Fig. 2c and Supplementary Movie 2). While aminoglycoside-treated cells presented almost perfect synchrony in growth arrest, DNA synthesis inhibitors gave rise to more heterogeneous cell behaviour. In case of ciprofloxacin, this heterogeneity showed to be even more pronounced at drug concentrations below the F10 level (Supplementary Fig. 4 and Supplementary Movie 3).

### Bacteriolytic antibiotics induce heteroresistance

**Figure 3:**
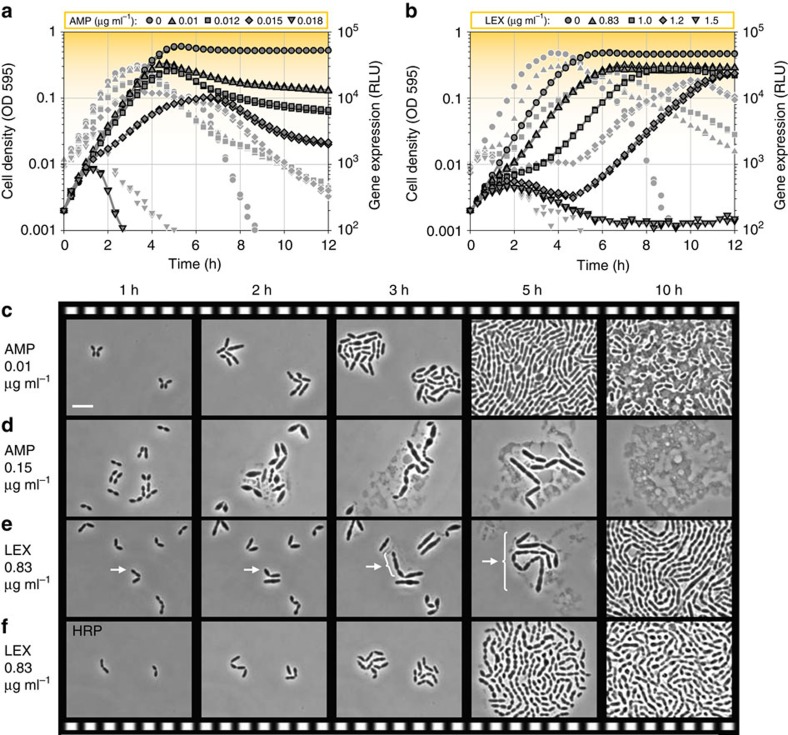
Growth-inhibition profiles of bacteriolytic antibiotics. (**a**,**b**) Plate reader assay sets in duplicates of *S. pneumoniae* D-PEP22 cells treated with a concentration series of ampicillin (AMP) (**a**) and cephalexin (LEX; **b**) measuring cell density (closed symbols) and gene expression (symbols without outline). Experiments were replicated at least three times. (**c**–**f**) Still images (phase contrast) after 1, 2, 3, 5 and 10 h of incubation of D-PEP22 cells growing on a semisolid surface containing 0.01 μg ml^−1^ ampicillin (**c**), 0.015 μg ml^−1^ ampicillin (**d**), 0.83 μg ml^−1^ cephalexin (**e**, arrows point to a cell developing the HRP) and the HRP of D-PEP22 also growing in the presence of 0.83 μg ml^−1^ cephalexin (**f**). Scale bar, 5 μm.

Commonly used antibiotics in clinical therapy include cell wall synthesis inhibitors that are also called bacteriolytic antibiotics (as a subcategory within bactericidal drugs) since they induce cell lysis^32^,^33^,^34^. Initial growth rates in the presence of β-lactam antibiotics were comparable to those of untreated cells, which is in line with our previous observations for bactericidal antibiotics (Fig. 3a,b). Interestingly, vancomycin seemed to impact growth cultures more promptly, and this drug is also attributed to be less bactericidal^35^ (for complete list see Supplementary Fig. 5). Antibiotics with bacteriolytic activity, with the example of ampicillin, showed that a minor increase in the applied drug concentration above a critical level was sufficient to cause cell lysis (Fig. 3a). Concentrations below this critical level allowed for uninhibited growth and merely led to decreased final cell densities and increased lysis during the stationary phase^36^. Single-cell analysis using time-lapse microscopy confirmed these observations and revealed that cells exhibited increased sizes and altered morphologies, such as lemon shapes, in line with a direct impairment of the cell wall synthesis machinery (Fig. 3c,d and Supplementary Movie 4)^33^,^37^.

In contrast to ampicillin, cultures treated with cephalexin showed the renewal of strikingly rapid cell growth after lag periods that increased with the applied drug concentration (Fig. 3b). Similar behaviours were also found with penicillin G and vancomycin (Supplementary Fig. 5). The observed lag periods might indicate that cells of the entire population needed time to adapt to the applied change of the environmental growth condition. An alternative explanation could be the presence of a population bifurcation into growing and non-growing cells. The phenomenon of antibiotic exposure resulting in reduced cell fractions that successfully grow out to form colonies has been previously described as heteroresistance^13^,^14^. Heteroresistance towards cell wall synthesis inhibitors in *S. pneumoniae* is classically identified via population analysis profiles (PAP) by plating cells in the presence of concentration series of antibiotics and subsequent CFU counting^14^. We carried out PAP and found indeed fewer CFUs emerging from exposure to higher bacteriolytic drug levels (Supplementary Fig. 5).

To characterize cells that spontaneously displayed decreased drug susceptibility, we isolated six colonies originating from plates with 1.5 μg ml^−1^ cephalexin (where only 1% of CFUs emerged compared with plates without antibiotics) and continuously cultivated cells in the presence of low amounts of cephalexin (0.83 μg ml^−1^). Remarkably, cells of these isolates readily grew at cephalexin concentrations that caused lags of the parental strain, which indicates that the decreased antibiotic susceptibility was inherited for several generations (Supplementary Fig. 6). We hypothesized that this might be caused by epigenetic inheritance since relatively high fractions of cells developed this heteroresistance phenotype (from here on HRP). Furthermore, re-culturing of HRP cells in antibiotic-free medium rendered the overall population more susceptible again, and plating in absence of antibiotics re-established wild type-like growth-inhibition profiles (Supplementary Fig. 6). To confirm an epigenetic mechanism, and to exclude reversible genomic rearrangements, we sequenced all six HRP strains, together with six corresponding cephalexin-susceptible strains that were obtained from plating cells of HRP strains without antibiotics. While two HRP strains showed identical genome sequences compared with the parental strain, four contained single point mutations; nevertheless, none of the mutations seemed likely to contribute to decreased drug susceptibility (Table 2). Furthermore, reversion strains contained the same point mutations as their corresponding HRP strain, and only in one case an additional point mutation had accumulated.

All resequenced genomes, including the one of the parental strain, gave evidence for new junctions at the *hsdS* gene, encoding the S subunit of a type I restriction-modification system. The *hsdS* locus was recently identified to undergo reversible genomic rearrangements, giving rise to distinct DNA methylation patterns that in turn result in different global gene expression profiles^38^. This mechanism was shown to significantly alter the expression of extracellular virulence factors, and therefore represented a potential candidate to also affect drug susceptibility. We treated a *hsdS* knockout strain with cephalexin and found identical inhibition profiles as with D-PEP22, demonstrating that *hsdS*-dependent gene expression regulation does not drive heteroresistance (Supplementary Fig. 7).

We investigated the phenomenon of heteroresistance also at the single-cell level by conducting time-lapse microscopy in the presence of cephalexin (0.83 μg ml^−1^). We found that most cells lysed, and only few cells grew out to form microcolonies; susceptible cells were thus not only inhibited but also killed in the course of the antibiotic treatment (Fig. 3e and Supplementary Movie 5). In contrast, cells originating from cultures of HRP strains readily grew out to form microcolonies in the presence of cephalexin (Fig. 3f and Supplementary Movie 5). Next, we focused on the dynamics underlying HRP development. In PAPs, higher drug levels resulted in fewer CFUs, and in our real-time experiments in liquid culture, treatment with higher antibiotic concentrations resulted in longer lag periods before subpopulations appeared (Fig. 3b). These observations could be explained by two possible mechanisms. Several phenotypes with different drug susceptibilities might co-exist, and therefore fewer pre-adapted cells survive an environmental shift to higher drug concentrations. Alternatively, the development of the HRP might be triggered by antibiotics, and since antibiotic treatment also induces lysis, a population equilibrium establishes in which the survival interval for cells to accomplish phenotype switching becomes more constrained at higher drug concentrations. We approached this question by analysing HRP cultures originating from a series of cephalexin concentration (0.68, 0.83, 1.0 and 1.2 μg ml^−1^) and found that cells of all cultures displayed identical decreases in antibiotic susceptibility (Supplementary Fig. 8). To test whether HRPs originating from treatment with different bacteriolytic antibiotics confer cross-protection, we exposed HRPs coming from penicillin G, vancomycin and cephalexin to drug concentrations that allowed for robust subpopulation emergence, and additionally to ampicillin at a level of full inhibition (ampicillin 0.018 μg ml^−1^, penicillin G 0.01 μg ml^−1^, vancomycin 0.18 μg ml^−1^ and cephalexin 1.0 μg ml^−1^). All cultures showed identical decreases in susceptibility (Supplementary Fig. 9). These findings indicate the existence of one unique HRP for all bacteriolytic antibiotics, which develops upon induction, and in competition with ongoing cell lysis. Ampicillin (in comparison with other bacteriolytic compounds) demonstrated poor induction of the HRP; however, heteroresisters nevertheless exhibited decreased susceptibility towards this antibiotic (Supplementary Fig. 9).

During time-lapse microscopy in the presence of cephalexin, cells inflated at midcell, the place where cell wall synthesis takes place in *S. pneumoniae*^39^, and eventually burst when exceeding a critical size (Fig. 3e). Cells of HRP populations also displayed increased sizes during cephalexin treatment (at concentrations that killed most cells of the parental populations); however, the inflation seemed to not exceed the critical limit. To test whether cell size plays a role in cephalexin susceptibility, we analysed the capsule mutant D39 *Δcps* that is known to feature smaller sizes^40^ (similar length but a reduced maximal width; 0.67±0.05 μm compared with 0.74±0.05 μm), and found indeed decreased cephalexin susceptibility and furthermore greatly diminished heteroresistance development (Supplementary Fig. 10). Nevertheless, time-lapse microscopy revealed that cell-to-cell variability in susceptibility towards cephalexin was still present in D39 *Δcps* (Supplementary Fig. 10 and Supplementary Movie 6). This result demonstrates that capsule expression renders pneumococci more susceptible to cell wall synthesis inhibitors and indicates that heteroresistance of *S. pneumoniae* towards bacteriolytic drugs might involve an epigenetic inheritance of factors influencing cell morphology^39^,^41^.

### Metabolic activity and heterogeneity potentiate resistance

**Figure 4:**
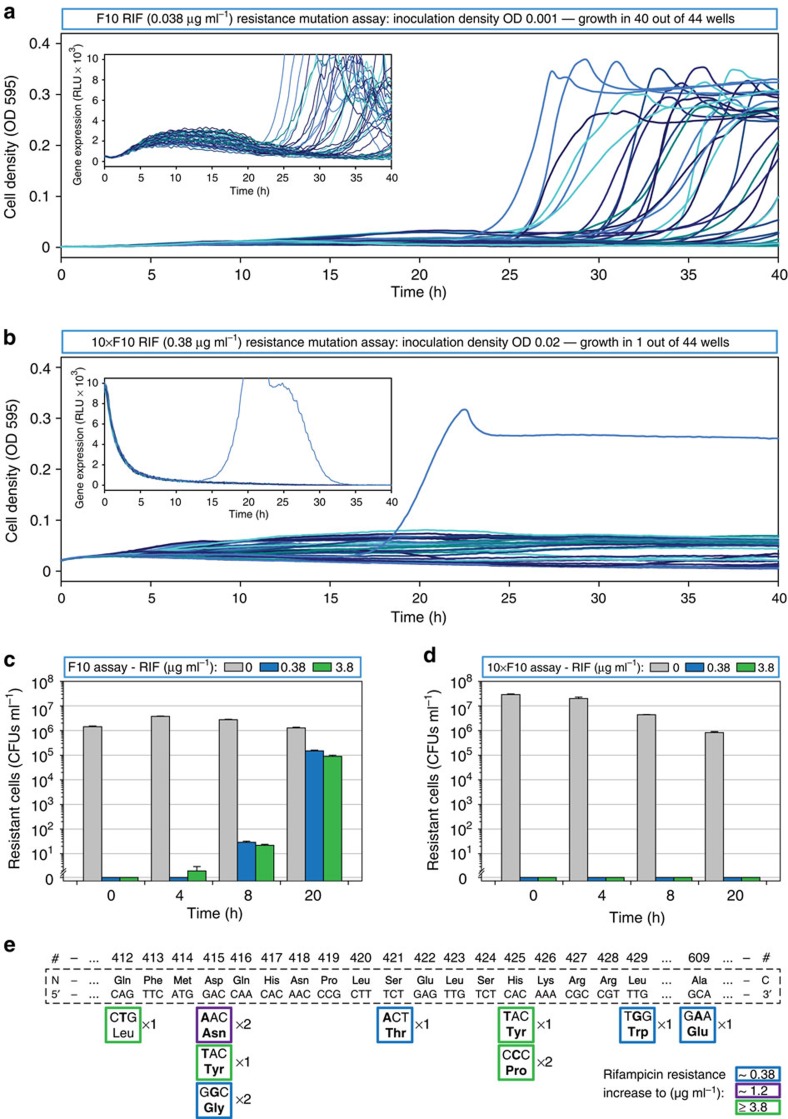
Rifampicin resistance mutation assay. (**a**,**b**) *S. pneumoniae* D-PEP22 populations seeded in 44 microtitre plate wells at an inoculation density of OD 0.001 in the presence of 0.038 μg ml^−1^ RIF (**a**) and at an inoculation density of OD 0.02 in the presence of 0.38 μg ml^−1^ RIF (**b**), followed over time by measuring cell density (OD595) and gene expression (RLU; insets). Note that results are shown on linear scales, which more clearly display the emergence of resistant populations. (**c**,**d**) Number of resistant cells (CFUs ml^−1^) after plating pooled samples of 44 microtitre plate wells (75 μl sampling volume per well per time point) originating from 0.038 μg ml^−1^ rifampicin (**c**) and 0.38 μg ml^−1^ rifampicin (**d**) treatments (duplicate assays of the ones shown in **a**,**b** based on the same pre-culture) directly into 0, 0.38, and 3.8 μg ml^−1^ RIF, respectively; average and s.e.m. of duplicates are shown. (**e**) Sequencing of *rpoB* of 12 isolates originating from spontaneously resistant populations during 0.038-μg ml^−1^ RIF treatment, with the relevant RpoB amino-acid sequence (bold) together with the codons inside the dashed frame and their position in italics above; below, point mutations, the increase in resistance they generate and their frequency of occurrence are indicated.

To test whether F10 concentrations support resistance evolution, as is presumed for antibiotic concentration ranges within the MSW, we monitored and quantified the emergence of spontaneously resistant populations in real time^42^,^43^. Rifampicin is an excellent candidate for such mutation assays because single point mutations, which may occur at various locations within the *rpoB* gene (coding for the β-subunit of RNA polymerase), are sufficient for resistance^44^,^45^. We first tested rifampicin at the F10 level (0.038 μg ml^−1^) with a low inoculation density of 4.5 × 10^5^ CFUs per culture (in a volume of 300 μl) and observed growth within 40 h in 40 out of 44 seeded populations (Fig. 4a). In a second assay, a rifampicin concentration of 10 × F10 (0.38 μg ml^−1^) was used to treat 20 times more cells as a seed (9 × 10^6^ CFUs), and only one resistant population arose within the observed time (Fig. 4b). Both assays were performed in parallel together with two duplicate assays (44 populations for each condition) that were sampled at regular intervals to evaluate the number of CFUs and the number of resistant cells in real time (Fig. 4c,d). While the shared pre-culture of all assays did not show any presence of resistant cells, they eventually appeared and increased in number during the F10 treatment (Fig. 4c). In the 44 cultures of the 10 × F10 sampling assay, no resistance development could be detected over the time course of the assay (Fig. 4d; see also Methods).

Twelve out of the forty spontaneously resistant populations from the F10 assay were analysed in more detail. All isolates showed levels of rifampicin resistance much higher than the F10 requirement^46^,^47^ (sufficient for growth in 10 × F10), and sequencing attributed them to nine individual point mutations inside *rpoB* (Fig. 4e). The difference in resistance development between the two applied rifampicin concentrations could, thus, not be explained by a reduced frequency of mutations conferring high resistance. In addition, the number of viable cells at F10 concentrations did not surpass the number in 10 × F10 assays before 20 h (Fig. 4c,d). A likely explanation is that at F10 concentrations, treated populations showed continuous gene expression activity compared with 10 × F10, where luminescence levels dropped rapidly. Interestingly, the single spontaneously resistant population that emerged in the 10 × F10 assay appeared at a relatively early time point, suggesting that the mutation event took place during pre-cultivation or at an early stage of rifampicin exposure, when cells still showed metabolic activity (Fig. 4b). Together, these results suggest that gene expression activity is a prerequisite for resistance development.

**Figure 5:**
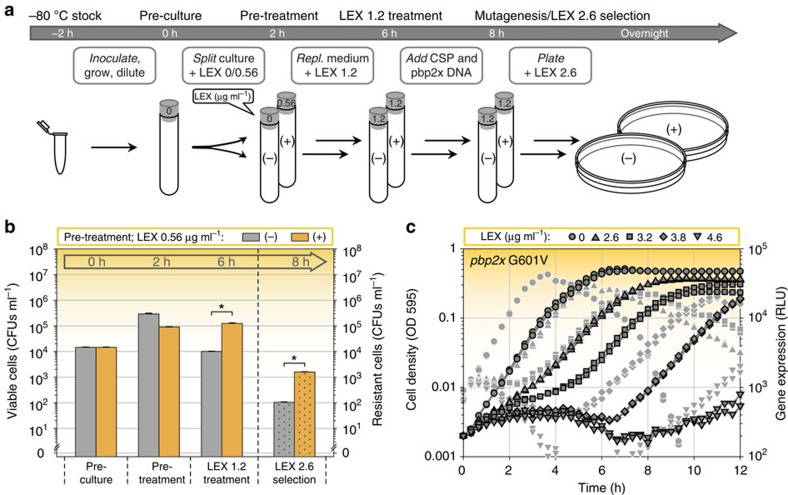
Cephalexin resistance accumulation assay. (**a**) Schematic representation of the assay, starting from a shared pre-culture that was divided into two pre-treatments, by adding no antibiotics (−) to one and 0.56 μg ml^−1^ cephalexin (+) (LEX) to the other. After 2 h of pre-treatment, both cultures were exposed to 1.2-μg ml^−1^ cephalexin for 4 h, followed by competence induction with 100 ng ml^−1^ competence-stimulating peptide (CSP). DNA encoding *pbp2x* G601V was provided, allowing for genetic resistance accumulation, and cells that successfully incorporated this resistance factor were selected on plates containing 2.6 μg ml^−1^ cephalexin. (**b**) Development of the viable cell count of the control (−) and the pre-treated (+) culture in the course of the assay; dotted bars indicate the number of CFUs after mutagenesis on plates containing 2.6 μg ml^−1^ cephalexin; average and s.e.m. of duplicates are shown; asterisks indicate significance (*P*<0.01, *t*-test). (**c**) Plate reader assay sets in duplicates of *S. pneumoniae* D-PEP22 *pbp2x* G601V cells treated with a concentration series of cephalexin, measuring cell density (closed symbols) and gene expression (symbols without outline). Experiments were replicated at least three times.

Finally, we wanted to determine whether the potential for resistance development towards bacteriolytic drugs (at concentrations that did not allow for HRP growth) was influenced by the observed heterogeneity in susceptibility towards cell wall synthesis inhibitors. To test this, we induced HRP development with a low concentration of cephalexin (0.56 μg ml^−1^) during 2 h and examined whether these populations would have an advantage over populations that did not experience such a pre-treatment. Indeed, after 4 h of cephalexin treatment at an inhibiting concentration of 1.2 μg ml^−1^, pre-treated cultures contained significantly more viable cells (12 × more CFUs, *P*<0.01, *t*-test) than control cultures (despite the fact that these cultures needed to compensate for a reduced CFU increase during the pre-treatment). Since spontaneous mutations towards bacteriolytic drug resistance occur rarely, which complicates statistical analysis, and at various loci^48^, which complicates their identification, we simulated the opportunity for genotypic resistance acquisition in a directed way via competence. When providing a DNA fragment coding for a mutated penicillin-binding protein (*pbp2x* G601V)^49^, we found significantly more resistant cells (15 × more CFUs, *P*<0.01, *t*-test) arising from pre-treated cultures upon selection with cephalexin (Fig. 5a,b). This result shows that heterogeneity in susceptibility to antibiotics, or in other words the presence of a fraction of cells with decreased epigenetic susceptibility, can potentiate the ability of a population to develop genotypic resistance. Remarkably, mutated strains showed again heteroresistance development at an increased concentration window compared with the wild type, demonstrating that resistance can build up in an iterative process via heteroresisters (Fig. 5c).

## Discussion

**Figure 6:**
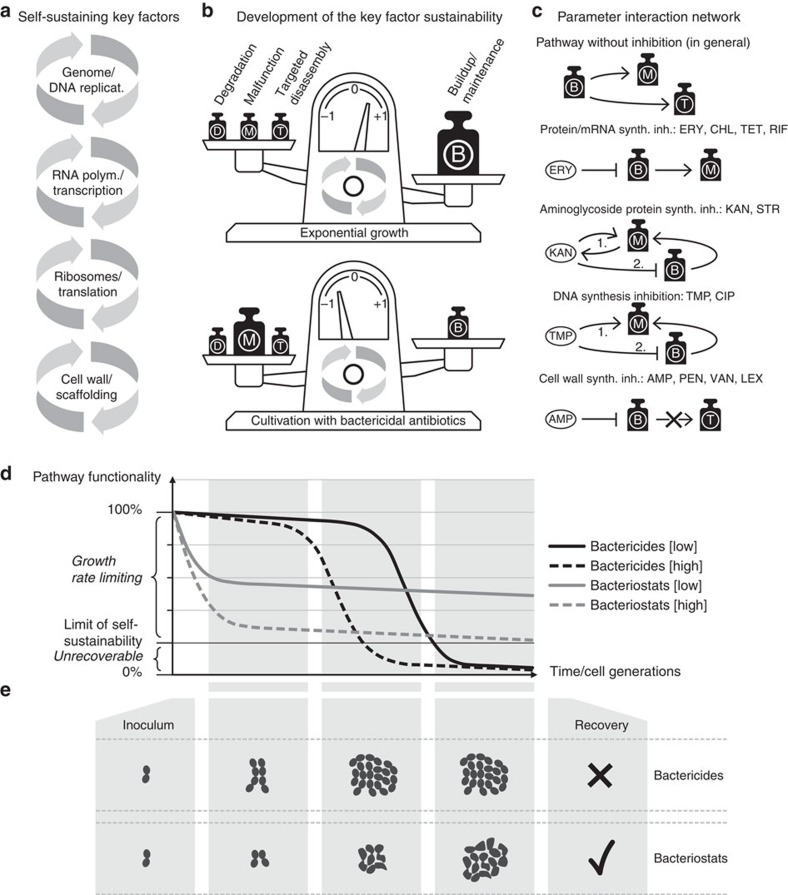
Network architecture of antibiotic growth inhibition and killing. (**a**) Essential pathways, such as DNA replication, contain key factors, such as the genome, that are self-sustaining in the sense that they cannot regenerate once they are lost (examples shown are non-exhaustive). (**b**) The key factor functionality can build up (B) by, for example, the replication or the repair of the genome but also decrease by degradation (D) and by self-inflicted damages of involuntary malfunctions (M), such as the incorporation of wrong nucleotides, and voluntary processes termed here targeted disassembly (T), such as the requirement to temporarily separate DNA strands to synthesize new genome copies. The combined effect of all parameters indicate whether the key factor functionality increases (growth), there is no net change (growth arrest), or whether it declines (damage). (**c**) Self-inflicted damages are in general dependent on ongoing buildup since higher synthesis activity leads to more synthesis mistakes and also requires more targeted disassembly. This parameter relationship is maintained in cells inhibited by bacteriostatic antibiotics. Bactericidal antibiotics in contrast disturb the parameter network by disproportionately increasing malfunctions (1) before the buildup machinery becomes arrested (2), or by blocking buildup without mediating a simultaneous downregulation of the targeted disassembly. (**d**) Bactericidal antibiotics (bactericides) do not initially inhibit the functionality of the targeted pathway but initiate processes that lead to the subsequent rapid loss of the key factor integrity. In contrast, the impact of bacteriostatic antibiotics (bacteriostats) is instantaneous; the remaining pathway functionality however stays above a critical level since inhibiting buildup also inhibits the driving force for malfunctions, and thus circumvents a loss of the key factor integrity. (**e**) Schematic representation of growth, morphology and recovery of bactericidal and bacteriostatic drug-treated cells.

Here we investigated the impact of antibiotic treatment of *S. pneumoniae*, with an emphasis on conditions that allow for the development of genotypic resistance. Our results show that the growth-inhibitory effect of antibiotics with bactericidal activity at MSW concentrations manifests with a time lag, not only for *S. pneumoniae* but also for *Escherichia coli* (Supplementary Fig. 11; note that the impact of trimethoprim on *E. coli* MC1061, classified as bacteriostatic, affected growth more promptly in comparison with *S. pneumoniae* D39, where the impact is classified as bactericidal). Moreover, evidence is provided showing that cell damage already occurs during this initial period of uninhibited growth (Figs 1g and 3d). It is known that bactericidal antibiotics often provoke damages in an indirect manner that requires cellular metabolic activity^50^,^51^,^52^,^53^,^54^,^55^. These damages threaten bacterial survival because essential pathways are targeted, and the functionality of essential pathways critically depends on key factors that share the characteristic of being indispensable for their own reproduction; one needs for example a functional genome to replicate genomes, functional ribosomes to translate ribosomal proteins and so on (Fig. 6a). The disturbance of metabolic processes by bactericidal antibiotics dramatically increases fatal malfunctions, such as DNA strand breaks or mistranslations, or it destabilizes a delicate balance between counteracting cellular mechanisms, such as cell wall synthesis and autolysin activity, and may ultimately result in the loss of the key factor integrity of the targeted pathway (Fig. 6b). In theory, if damages impair the functionality of the very same cell components that drive the damaging (among others), then negative feedback is generated that reduces the number of new damages for increasingly damaged cells. Instantaneous back coupling should finally result in a stable state of cell perturbation that is likely to be reversible. In case of delayed or missing feedback on the other hand, as demonstrated by the time lag of growth inhibition with bactericidal antibiotics, the severity of damages can reach levels that go beyond recoverability (Fig. 6c–e). The instantaneous impact of an antimicrobial compound on the functionality of the targeted pathway thus represents a potential contradiction for bactericidal activity.

In the context of resistance development, the timing aspect of antibiotic impact is also highly relevant. MSWs are not only concentration ranges in which resistance mutations become advantageous but also the time frame for cells to acquire mutations, therefore spanning the window from initial growth inhibition until extinction of a susceptible cell population. Delayed growth inhibition reduces the period in which mutants have a selective growth advantage, and cell death reduces the period for mutants to arise. A drug that maximally limits the potential for resistance development should consequently induce cell damages to a lethal extent when growth is not affected yet. Aminoglycosides and β-lactam antibiotics come closest to this requirement. Furthermore, the MSW of an ideal drug should span a narrow concentration range; this requirement comes closest to our findings for cell wall synthesis inhibitors that poorly induce HRP transition (such as ampicillin). As a word of caution, some antibiotics are contraindicative for certain therapies, such as bacteriolytic drugs in case of bacteremia that risk triggering a septic shock upon the release of bacterial cell components^56^,^57^.

Bacteriostatic drugs do not kill bacteria directly but lead to lasting detention periods of growth rate limitation, which generally promote resistance development (Fig. 6d,e). The emerging cell morphologies indicate a perturbed homeostasis (Fig. 6e). Remarkably, inhibited cells retained metabolic activity, and mutation assays with rifampicin linked this activity to resistance development (Fig. 4). The importance of gene expression activity might rely on the fact that spontaneously acquired resistance traits need to be expressed before resistance takes effect. Alternatively, mutation rates might increase with gene expression activity^58^. For bacteria that are inhibited by bactericidal antibiotics, the proposed requirement of gene expression activity represents a veritable dilemma since this activity could drive both cell death and the only way for rescue. Furthermore, the involvement of persisters in resistance development would depend on whether these cells are metabolically active or not^15^,^59^. Nevertheless, epigenetic mechanisms can play a role in resistance evolution, as demonstrated here by comparing cephalexin resistance accumulation with or without a stepwise exposure to the antibiotic, and thus with or without previous heteroresistance induction (Fig. 5). Cell-to-cell variability in susceptibility to antibiotics, which presumably provides a subset of cells with an expanded window for mutagenesis, occurs not only in *S. pneumoniae* but also in unrelated bacteria, as for example in rifampicin-inhibited *E. coli* (Supplementary Fig. 12 and Supplementary Movie 7). Together, these results suggest that both metabolic activity and heterogeneity in antibiotic susceptibility are important factors for resistance development. Rapid gene expression shutdown and limited cell-to-cell variability are preferable characteristics that should be taken into consideration when screening for new drugs or determining drug dosage. To this effect, our experimental approach provides a framework for testing novel antimicrobial compounds on their prospects of delivering lasting effectiveness against fast-evolving bacterial pathogens.

## Methods

### Strains and growth conditions

*S. pneumoniae* D-PEP22, a D39 derivate strain that constitutively expresses firefly luciferase from a synthetic promoter (driven by the only housekeeping sigma factor of the pneumococcus) was used throughout^26^. Firefly luciferase has a reported half-life of 3 min in *S. pneumoniae*^27^, and luminescence therefore gives real-time information on the level of gene expression activity. *S. pneumoniae* D39 MK119 expressing *hlpA-mKate2*, a fusion of the DNA-associated histone-like protein A with the fluorescence protein mKate2, was used for time-lapse microscopy in the presence of ciprofloxacin at a concentration below the F10 level^60^. *S. pneumoniae* D39 *ΔhsdS* (Domenech, Kjos and Veening, unpublished) and *Δcps* (Jørgensen and Veening, unpublished) were used for heteroresistance analysis. *E. coli* experiments were performed with strain MC1061 (ref. 61).

*S. pneumoniae* cells were grown in C+Y medium^62^ (pH 6.8) supplemented with 0.5 μg ml^−1^ D-luciferine at 37 °C. Note that competence does not develop at this pH^23^. *E. coli* MC1061 cells were grown in LB medium at 37 °C. Pre-cultures for all experiments were obtained by a standardized protocol, in which previously exponentially growing cells from −80 °C stocks were diluted to OD (600 nm) 0.005 and grown until OD 0.1 (in a volume of 2 ml) in tubes that allow for direct OD measurements^26^. To determine the number of CFUs, *S. pneumoniae* D-PEP22 cells were plated inside Columbia agar supplemented with 3% (v v^−1^) sheep blood and incubated overnight at 37 °C.

### Antibiotics

The classification of antibiotics into bacteriostatic and bactericidal compounds is strain-specific, and the impact ratio of growth inhibition versus killing may even depend on environmental growth conditions and on the applied drug concentration (sometimes resulting in inverse relationships, such as slower killing at higher drug levels, the so-called paradoxical effect)^63^. For simplicity, we refer to antibiotics here as ‘bacteriostatic’, ‘bactericidal’ or ‘bacteriolytic’ when their reported impact on *S. pneumoniae* is predominantly bacteriostatic, bactericidal or bacteriolytic. Two dozen antibiotics were initially considered. The list was finally reduced because of target redundancies, and because of similar results obtained by pairs of antibiotics during initial experiments (such as streptomycin/gentamicin or ampicillin/methicillin). Furthermore, nalidixic acid was excluded because *S. pneumoniae* D39 is naturally resistant to this compound, and spectinomycin could not be used because D-PEP22 contains a resistance marker for this antibiotic. Our study finally included the following 12 antibiotic compounds (purchased from Sigma-Aldrich): erythromycin (733.93 g mol^−1^), chloramphenicol (323.13 g mol^−1^), tetracycline hydrochloride (480.90 g mol^−1^), rifampicin (822.94 g mol^−1^), kanamycin sulfate (582.58 g mol^−1^), streptomycin sulfate salt (728.69 g mol^−1^), trimethoprim (290.32 g mol^−1^), ciprofloxacin (331.34 g mol^−1^), ampicillin sodium salt (371.39 g mol^−1^), penicillin G sodium salt (356.37 g mol^−1^), vancomycin hydrochloride (1485.71 g mol^−1^) and cephalexin (347.39 g mol^−1^). Note that pneumococci (and also many other Gram-positive bacteria) show a relatively high intrinsic resistance towards currently available aminoglycoside drugs, disqualifying these compounds from antibiotic therapy. However, aminoglycosides are regularly used against infections caused by Gram-negative pathogens, and we therefore included this important antibiotic family in our comprehensive study.

Killing mechanisms of the bactericidal drugs tested here are well studied and driven by cellular metabolic activity. Trimethoprim-treated cells, for example, compensate the lack of sufficient dTTP supply (because of the inhibition of dihydrofolate reductase) by incorporating dUTP in ongoing rounds of replication, which is sequentially processed and results in single-strand breaks^53^. Ciprofloxacin inhibits, among others, the re-ligation of double-strand cuts by DNA gyrase after relaxation of positive supercoils^54^. In case of aminoglycosides, translation activity is enduringly blocked after mistranslated membrane proteins allow for an increased drug influx, and high intracellular doses of the polycationic antibiotic build up that cannot be disposed of^52^. For bacteriolytic drugs, killing is mediated by an unbalance between inhibited cell wall synthesis in relation to ongoing autolysin activity^32^.

Antibiotic concentrations in dose–response studies were annotated in μg ml^−1^ (which can readily be translated to molar values). Standard dilution series were calculated by splitting one order of magnitude into 12 steps of similar size on a log scale. This resulted in the values 1, 1.2, 1.5, 1.8, 2.2, 2.6, 3.2, 3.8, 4.6, 5.6, 6.8, 8.3 and 10. Each step in this dilution series increases the applied concentration by ∼20% compared with the previous value. Note that there may be variations in the absolute amount of active drug molecules in antibiotic stock solutions because of differences between production batches, and also because of measurement errors in the preparation process. The relative drug amount within an applied concentration series (derived from the same stock solution) in contrast is more precise and merely depends on volume deviations of the pipetting.

MIC values were determined via broth microdilution^28^. Microtitre plate cultures (described below) in quadruplicate were exposed to concentration series of each antibiotic and were incubated for 16 h (Supplementary Figs 1 and 5). According to the definition, MICs represent the upper concentration of the threshold between growth and no-growth, as observed with the unaided eye, within a concentration series of an antibiotic compound. OD 0.05 was chosen as the threshold of visual detectability.

### Microtitre plate reader assays

Costar 96-well plates (white, clear bottom) with a total assay volume of 300 μl per well were inoculated to the designated starting OD value. Microtitre plate reader experiments were performed using a TECAN infinite pro 200 plate reader (Tecan Group) by measuring every 10 min with the following protocol: 5 s shaking, OD (595 nm) measurement with 25 flashes, luminescence measurement with an integration time of 1 s. Plate reader assays proved to represent a straightforward method for analysing growth and gene expression profiles of pneumococci in a high-throughput manner. *S. pneumoniae* is particularly well suited for this technology since the organism is a microaerophile that does not require oxygen.

Some considerations for eliminating technical biases of plate reader experiments needed to be taken into consideration, enabling accurate data visualization using logarithmic scales. In our particular experimental set-up, OD measurements within the same well fluctuated over time in the range of 0.0003. This intrinsic mistake allowed for sufficient accuracy when measuring OD values of 0.002, which was generally used as inoculation density. This value is low enough for cells to grow during at least 4 h in the exponential phase, while at the same time it is high enough to allow for the detection of immediate lysis of cells from the inoculum. The difference in OD between wells of identical content varied to a much higher degree than the same well over time; a likely explanation for this phenomenon can be found in minor material irregularities within 96-well plates. It is consequently more accurate to determine individual OD blanks for each well instead of determining one average blank for all wells. The known OD value of the pre-culture and the dilution coefficient of the inoculum were used to back-calculate individual blanks. Note that the term ‘growth’ was mostly used in the sense of biomass increase, and thus cellular anabolic activity, detected by increasing OD values. While continuously exponentially increasing OD values allow for straightforward conclusions on growth of most cells within a culture, slowed OD increases or lags in the OD increase may have several causations (see Results). Bioluminescence in our experimental set-up showed no background levels but fluctuations in the range of 20 relative luminescence units (RLU, a.u.). D-PEP22 luciferase expression allowed for distinguishable luminescence signals within four orders of magnitude. Note that the detection limit for (uninhibited) D-PEP22 cells via bioluminescence was more than one order of magnitude higher than the detection limit for the cell density reads these cells generated. This difference in resolution is also the reason why small amounts of metabolically active cells, within overall repressed populations, could be detected via luminescence before these subpopulations dominated OD reads. Since, to the best of our knowledge, useful statistical tests do not exist for cell density measurements, we show replicate data of single experiments, and furthermore always include controls. Note that all experiments were independently repeated at least three times.

### Microscopy

Cells for fluorescence microscopy with DNA staining were obtained by spinning down 0.9 ml of culture and re-suspension in PBS containing 2 μg ml^−1^ Hoechst. After 10 min incubation at room temperature, cells were washed with PBS, and 1 μl of the cell preparation was spotted on a PBS–polyacrylamide (10%) slide inside a Gene Frame (Thermo Fisher Scientific) and sealed with the cover glass to guaranty stable conditions during microscopy. A Nikon Ti-E microscope equipped with a CoolsnapHQ2 camera and an Intensilight light source was used. Images of Hoechst-fluorescing cells were taken with the following protocol and filter settings: 0.3 s exposure for phase contrast, 0.5 s exposure for fluorescence at 340–380 nm excitation via a dichroic mirror of 400 nm and an emission filter of 435–485 nm.

Time-lapse microscopy in the presence of antibiotics was carried out by spotting pre-cultured cells on 10% polyacrylamide slides inside Gene Frames as described above. The polyacrylamide slides were prepared with the growth medium, and were furthermore incubated during 2 h in growth medium containing the designated antibiotic at F10 concentrations. Minor concentration adjustments were applied for some antibiotics to obtain F10 growth kinetics in the semisolid environment, which might be due to changes in pharmacodynamics (compared with liquid culture), or due to variations in antibiotic saturation of the polyacrylamide slide. The following concentrations were applied: erythromycin 0.068 μg ml^−1^, chloramphenicol 1.5 μg ml^−1^, tetracycline 0.32 μg ml^−1^, rifampicin 0.026 μg ml^−1^, kanamycin 220 μg ml^−1^, streptomycin 56 μg ml^−1^, trimethoprim 0.83 μg ml^−1^ and ciprofloxacin 0.83 μg ml^−1^ (and 0.68 μg ml^−1^ in case of MK119 cells). Temperature during microscopy was controlled with an Okolab climate incubator, and phase contrast images of cells were taken as described above every 10 min during 20 h at 37 °C. Images of mKate2 fluorescing cells (MK119) were taken every 20 min with 2 s exposure and a filter set of 560–600 nm for excitation via a dichroic mirror of 605 nm and emission of >615 nm (longpass filter). The first still frame of a time-lapse experiment is annotated as 1 h into the cultivation start (01:00); 1 h was the time required for reaching stable conditions inside the microscopy slide that allowed for automated recording.

Cell sizes of D-PEP22 and D39 *Δcps* were analysed with MicrobeTracker^64^.

### Sequencing of heteroresisters

D-PEP22 HRP and reversion strains for genome sequencing were grown in 4 ml cell culture to OD 0.2, followed by genomic DNA isolation according to standard protocol. HRP strains were cultivated in the presence of 0.83 μg ml^−1^ cephalexin to maintain the less drug-susceptible phenotype. Sequencing of DNA was performed with an Illumina MiSeq machine, using 250 nucleotide paired-end reads. Sequencing reads from each strain underwent quality control via the FastQC platform^65^ and were subsequently processed using Trimmomatic^66^. The *breseq* computational pipeline^67^ was used to detect single-nucleotide polymorphisms, indels and new junctions, as compared with the reference *S. pneumoniae* D39 genome^21^. Note that the term ‘heteroresistance’, in case of *Mycobacterium tuberculosis*^68^, is used to describe cell-to-cell variability in drug susceptibility of mixed populations, in contrast to parental populations as in the case of *S. pneumoniae* heteroresistance towards cell wall synthesis inhibitors^14^.

### Analysis of rifampicin- and cephalexin-resistant mutants

In rifampicin mutation assays, spontaneously resistant populations only appeared after prolonged incubation because mutants likely arise as single cells that need to divide multiple times before generating detectable cell density reads. On the basis that OD 0.1 contains 1.5 × 10^8^ CFUs ml^−1^, a single D-PEP22 cell requires ∼13 h to develop this cell density in a volume of 300 μl when assuming fast doubling times of 30 min. During pre-cultivation (or −80 °C stock preparation), mutation events may already occur that can give rise to resistant populations appearing at early time points (13–17 h) of assays in several of the seeded wells; in this scenario, the same mutation is found in cells of all of the early emerging populations, and can thus be traced back to a singular mutation event before the subdivision into individual cultures. Resistant populations that appear at later time points, especially when growing fast, are in contrast more likely to have developed in the course of the mutation assay. Note that the higher inoculation density of the 10 × F10 assay (compared with the F10 assay) was chosen to allow for similar amounts of viable cells, that could potentially gain resistance, until ∼20 h into the assay.

Using the luciferase-expressing strain D-PEP22 provided an additional advantage because luminescence allowed for an easy distinction between spontaneously resistant populations versus contaminations that are prone to appear in assays requiring long incubation times. Spontaneously resistant populations from rifampicin treatment (0.038 μg ml^−1^) were re-streaked without antibiotics, single colonies were isolated, grown in liquid culture and analysed in plate reader assays in the presence of a concentration series of rifampicin. The level of gained resistance was determined in relation to wild type D-PEP22 cultures. To identify mutations conferring increased rifampicin resistance, *rpoB* was analysed by Sanger sequencing with the primer 5′-GTGCGGTTGGTGAATTGCTTG-3′.

The *pbp2x* G601V DNA was prepared by exchanging codon 601 (GGA) with the triplet GTT via PCR assembly of upstream and downstream PCR products that overlapped 25 base pairs around the mutated site. Mutations in resistant strains were verified by Sanger sequencing with the primer 5′-GGCAAGATTCTTCCCTTCTTC-3′.

## Additional information

**How to cite this article:** Sorg, R. A. & Veening, J.-W. Microscale insights into pneumococcal antibiotic mutant selection windows. *Nat. Commun.* 6:8773 doi: 10.1038/ncomms9773 (2015).

## Supplementary Material

Supplementary Information Supplementary Figures 1-12

Supplementary Movie 1 Time-lapse microscopy in the presence of bacteriostatic antibiotics. Phase-contrast microscopy of a time-lapse experiment of *S. pneumoniae* D-PEP22 cells growing on a semi-solid surface containing F10 concentrations of the bacteriostatic antibiotics erythromycin (ERY, top left), chloramphenicol (CHL, top right), tetracycline (TET, bottom left), and rifampicin (RIF, bottom right). The control assay without antibiotics can be found in Supplementary Movie 4 (Control, top left). The first still frame of time-lapse experiments is annotated as one hour into the cultivation start (01:00); one hour was the time required for reaching stable conditions inside the microscopy slide that allow for automated recording.

Supplementary Movie 2 Time-lapse microscopy in the presence of bactericidal antibiotics. Phase-contrast microscopy of a time-lapse experiment of *S. pneumoniae* D-PEP22 cells growing on a semi-solid surface containing F10 concentrations of the bactericidal antibiotics kanamycin (KAN, top left), streptomycin (STR, top right), trimethoprim (TMP, bottom left), and ciprofloxacin (CIP, bottom right).

Supplementary Movie 3 Time-lapse microscopy in the presence of ciprofloxacin at a concentration below F10. Overlay of phase-contrast and fluorescence microscopy of a time-lapse experiment of *S. pneumoniae* MK119 cells (that express the DNA associated red-fluorescing fusion protein HlpA-mKate2) growing on a 10% polyacrylamide slide that was incubated in C+Y medium containing 0.68 μg ml^−1^ ciprofloxacin. High levels of fluorescence indicate on-going gene expression activity because existing pools of mKate2 bleach rapidly. Fluorescence intensity and its distribution furthermore give information about DNA abundance and nucleoid morphology inside the DNA synthesis-inhibited cells.

Supplementary Movie 4 Time-lapse microscopy in the presence of ampicillin. Phase-contrast microscopy of a time-lapse experiment of *S. pneumoniae* D-PEP22 cells growing on a semi-solid surface containing ampicillin at a concentration of 0 μg ml^−1^ (Control, top left), 0.01 μg ml^−1^ (top right), 0.015 μg ml^−1^ (bottom left), and 0.022 μg ml^−1^ (bottom right).

Supplementary Movie 5 Time-lapse microscopy in the presence of cephalexin. Phase-contrast microscopy of a time-lapse experiment of *S. pneumoniae* D-PEP22 wild-type cells growing on a semi-solid surface containing 0.83 μg ml-1 cephalexin (top left) and without antibiotics (Control, bottom left), and the D-PEP22 heteroresistance phenotype (HRP) also growing in presence of 0.83 μg ml-1 cephalexin (HRP, top right) and without antibiotics (HRP — Control, bottom right).

Supplementary Movie 6 Time-lapse microscopy of *S. pneumoniae* D39 *Δcps* in the presence of cephalexin. Phase-contrast microscopy of a time-lapse experiment of *S. pneumoniae* D39 *δcps* cells growing on a semi-solid surface containing 1.0 μg ml-1 cephalexin.

Supplementary Movie 7 Time-lapse microscopy of *E. coli* MC1061 in the presence of rifampicin. Phase-contrast microscopy of a time-lapse experiment of *E. coli* MC1061 cells growing on a 10% polyacrylamide slide that was incubated in LB medium containing 10 μg ml^−1^ rifampicin. To exclude a contamination of the MC1061 −80°C stock as origin of the observed cell-to-cell heterogeneity in rifampicin sensitivity, the MC1061 culture was re-streaked and cells from an isolated single colony were used.

## Figures and Tables

**Table 1:** List of antibiotics and their classification in *S. pneumoniae*.

**Name**	**Abbr.**	**Family**	**Inhibited pathway**	**Target (enzyme activity)**	**Classification** *	**MIC**† **[μg ml**^**−1**^]	**F10**‡ **[μg ml**^**−1**^]
Erythromycin	ERY	Macrolides	Protein synthesis	Aminoacyl translocase	Bacteriostatic	0.056	0.068
Chloramphenicol	CHL	—	Protein synthesis	Peptidyl transferase	Bacteriostatic	2.2	2.6
Tetracycline	TET	Tetracyclines	Protein synthesis	Aminoacyl-tRNA access	Bacteriostatic	0.26	0.32
Rifampicin	RIF	Rifamycins	mRNA synthesis	RNA polymerase	Bacteriostatic	0.032	0.038
Kanamycin	KAN	Aminoglycosides	Protein synthesis	30S subunit/mistranslations	Bactericidal	150	180
Streptomycin	STR	Aminoglycosides	Protein synthesis	30S subunit/mistranslations	Bactericidal	32	56
Trimethoprim	TMP	—	DNA synthesis	Dihydrofolate reductase	Bactericidal	1.0	1.8
Ciprofloxacin	CIP	Quinolones	DNA synthesis	Topoisomerase (type-II)	Bactericidal	1.2	1.5
Ampicillin	AMP	Penicillins	Cell wall synthesis	Transpeptidase (PBPs)	Bacteriolytic	0.018	—
Penicillin G	PEN	Penicillins	Cell wall synthesis	Transpeptidase (PBPs)	Bacteriolytic	0.015	—
Vancomycin	VAN	Glycopeptides	Cell wall synthesis	D-alanyl-D-alanine access	Bacteriolytic	0.22	—
Cephalexin	LEX	Cephalosporins	Cell wall synthesis	Transpeptidase (PBPs)	Bacteriolytic	1.5	—

^*^Colour code for antibiotic classifications (applied in figures): bacteriostatic in blue; bactericidal in red; and bacteriolytic in yellow.

^†^MICs, as determined by broth microdilution^28^.

^‡^F10 concentrations: drug amount that limits OD increase of treated cultures to a factor 10 within 10 h of cultivation.

**Table 2:** Genome-wide resequencing of heteroresisters.

**Strain #**	**HRP**	**Reversed to wild type phenotype**
1	=D-PEP22	=D-PEP22
2	glmS* V168G	*glmS* V168G
3	*glmS* G138S	*glmS* G138S
4	*glpK*† V343A	*glpK* V343A
5	=D-PEP22	*fucU*‡ N75N
6	Intergenic	Intergenic

HRP, heteroresistance phenotype.

^*^glmS: glucosamine–fructose-6-phosphate aminotransferase.

^†^*glpK*: glycerol kinase.

^‡^*fucU*: fucose operon FucU protein.
