# Supplementary material for: Microscale insights into pneumococcal antibiotic mutant selection windows
Source: Nat Commun. 2015 Oct 30;6:8773. doi: 10.1038/ncomms9773 (PMC4632196; doi:10.1038/ncomms9773)
Supplement: Supplementary Information — Supplementary Figures 1-12 [file ncomms9773-s1.pdf]

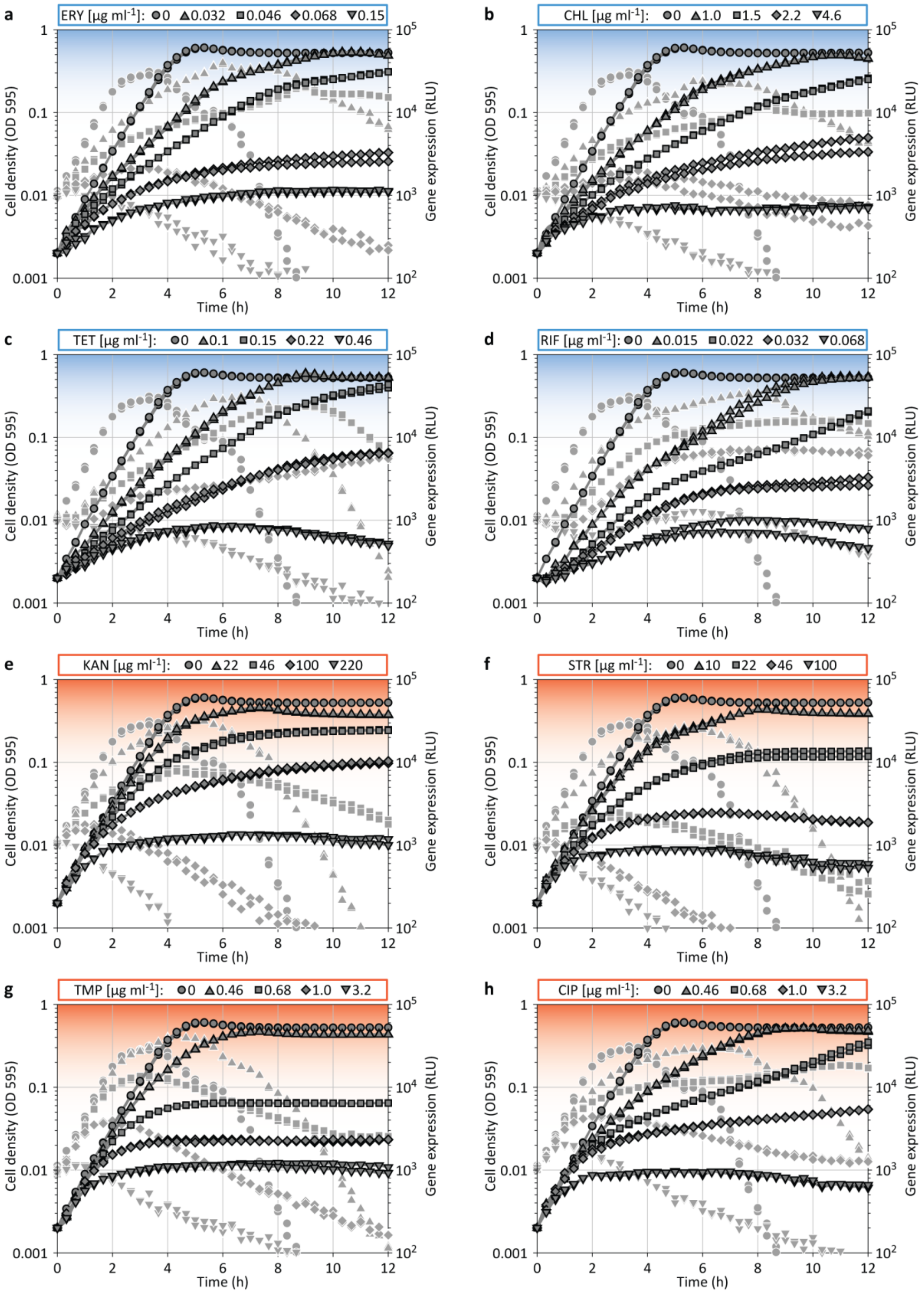

Supplementary Figure 1 | Growth-inhibition profiles of bacteriostatic and bactericidal drug treatment. a-h, Plate reader assay sets in duplicates measuring cell density (closed symbols) and gene expression (symbols without outline) of *S. pneumoniae* D-PEP22 growing in the presence of concentration series of the bacteriostatic antibiotics erythromycin (ERY) (**a**), chloramphenicol (CHL) (**b**), tetracycline (TET) (**c**), and rifampicin (RIF) (**d**), and the bactericidal antibiotics kanamycin (KAN) (**e**), streptomycin (STR) (**f**), trimethoprim (TMP) (**g**), and ciprofloxacin (CIP) (**h**).

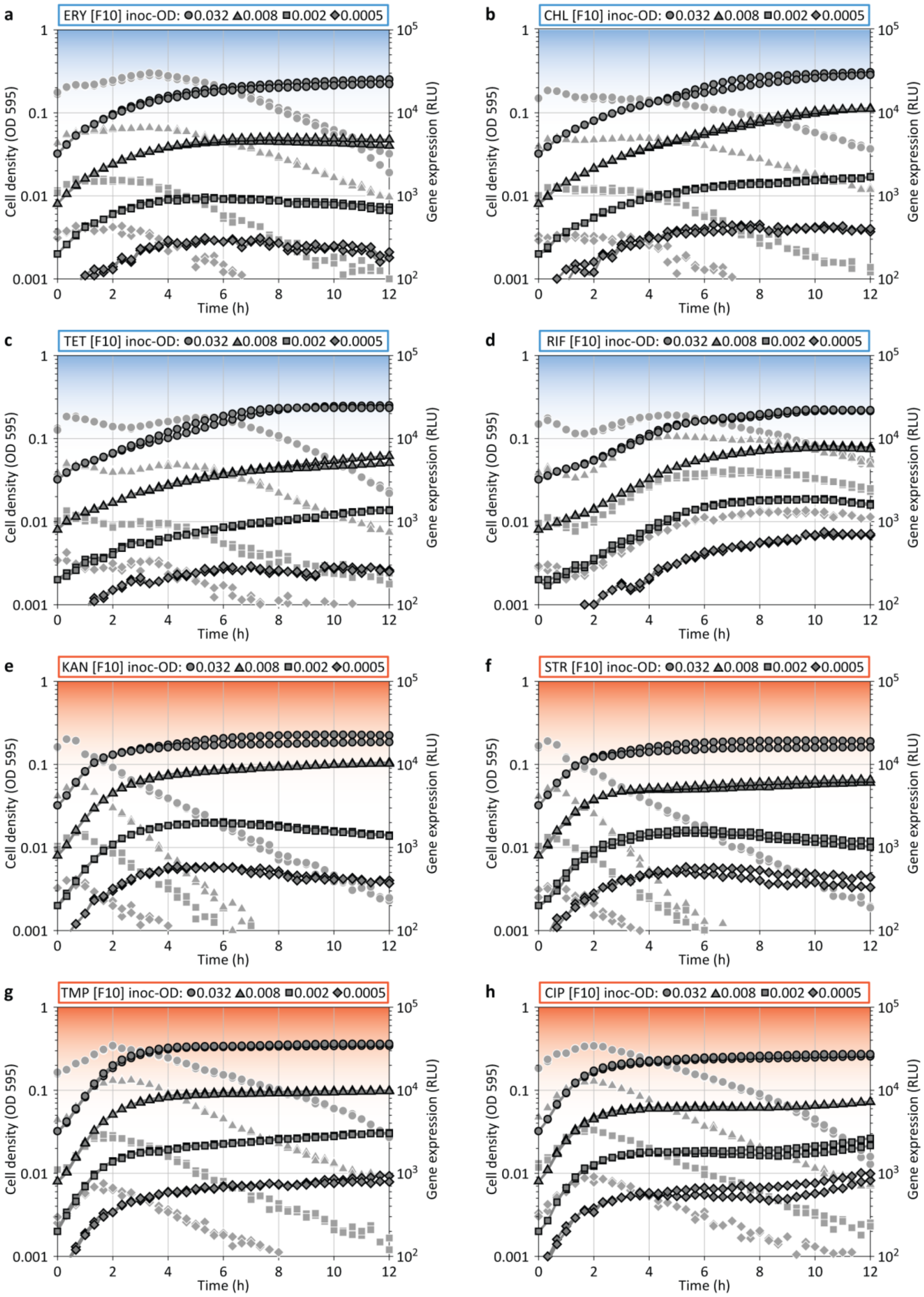

Supplementary Figure 2 | Impact of F10 concentrations on different inoculation cell densities. a-h, Plate reader assay sets in duplicates measuring cell density (closed symbols) and gene expression (symbols without outline) of a series of inoculation densities of *S. pneumoniae* D-PEP22 growing in the presence of F10 concentrations of the bacteriostatic antibiotics erythromycin (ERY) (**a**), chloramphenicol (CHL) (**b**), tetracycline (TET) (**c**), and rifampicin (RIF) (**d**), and the bactericidal antibiotics kanamycin (KAN) (**e**), streptomycin (STR) (**f**), trimethoprim (TMP) (**g**), and ciprofloxacin (CIP) (**h**).

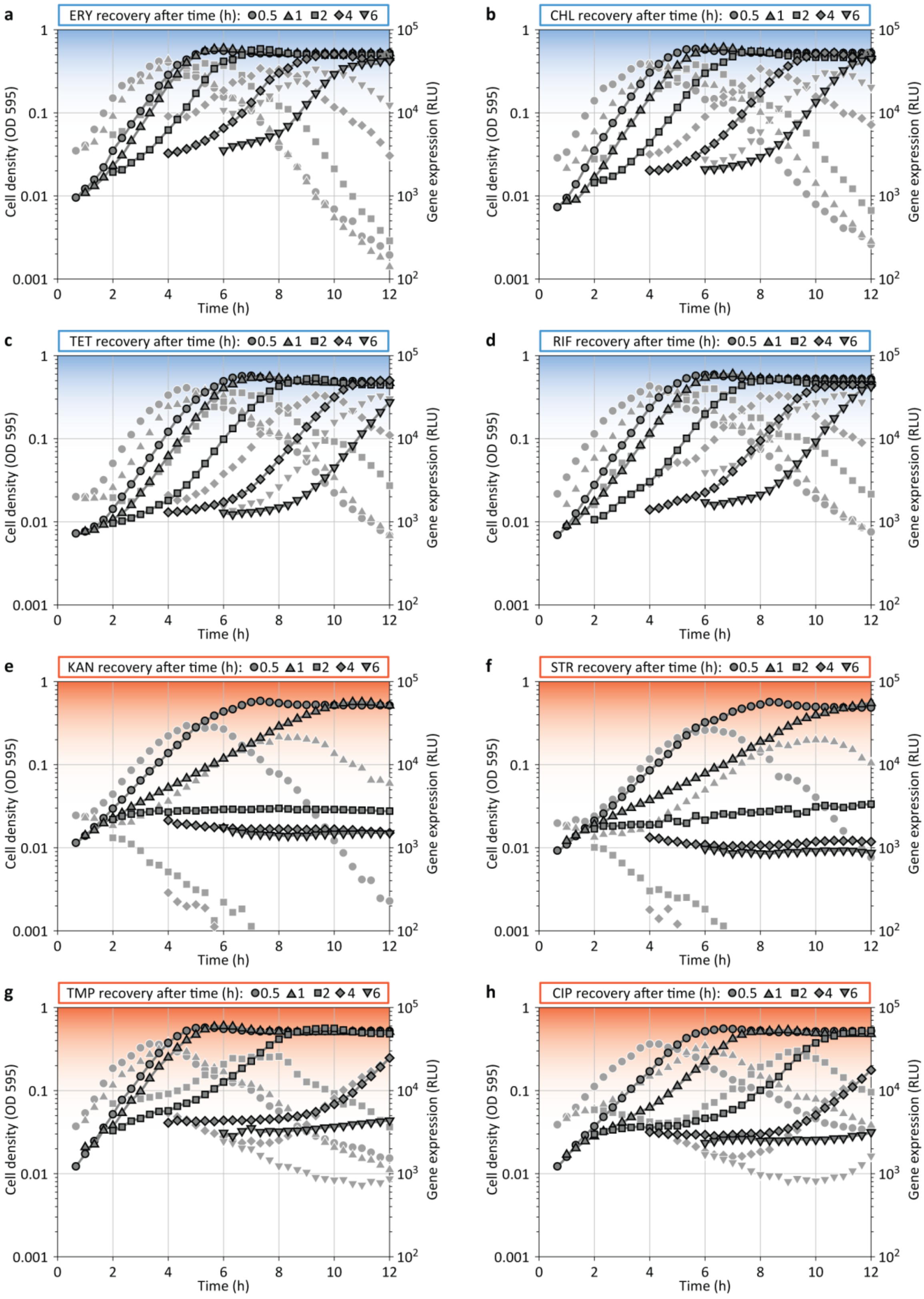

Supplementary Figure 3 | Recovery assay of bacteriostatic and bactericidal drug-treated cells. a-h, Plate reader assays measuring cell density (closed symbols) and gene expression (symbols without outline) of *S. pneumoniae* D-PEP22 recovering from 0.5, 1, 2, 4, and 6 hours of antibiotic treatment. Cells originated from two pooled microtiter plate cultures (per time point) that were exposed to F10 concentrations of erythromycin (ERY) (**a**), chloramphenicol (CHL) (**b**), tetracycline (TET) (**c**), rifampicin (RIF) (**d**), kanamycin (KAN) (**e**), streptomycin (STR) (**f**), trimethoprim (TMP) (**g**), and ciprofloxacin (CIP) (**h**) (see Fig. 1); the drug-containing medium was replaced by medium without antibiotics.

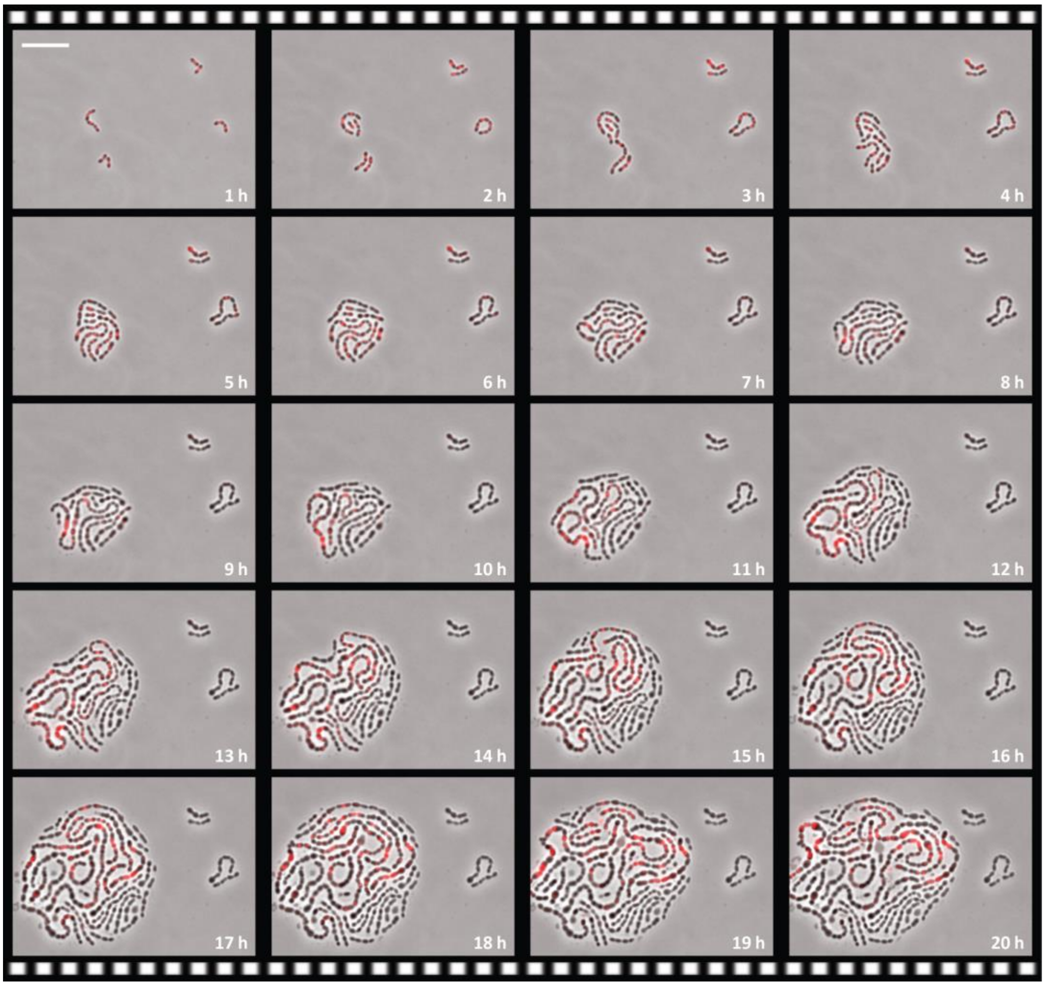

Supplementary Figure 4 | Time-lapse microscopy of ciprofloxacin treatment at a concentration below F10. Still images (overlay of phase-contrast and fluorescence microscopy) of a time-lapse experiment of *S. pneumoniae* MK119 cells (that express the DNA associated red-fluorescing fusion protein HlpA-mKate2) growing on a 10% polyacrylamide slide that was incubated in C+Y medium containing 0.68 µg ml^{-1} ciprofloxacin. High levels of fluorescence indicate ongoing gene expression activity because existing pools of mKate2 bleach rapidly. Fluorescence intensity and its distribution furthermore give information about DNA abundance and nucleoid morphology inside the DNA synthesis-inhibited cells. Scale bar is 10 µm.

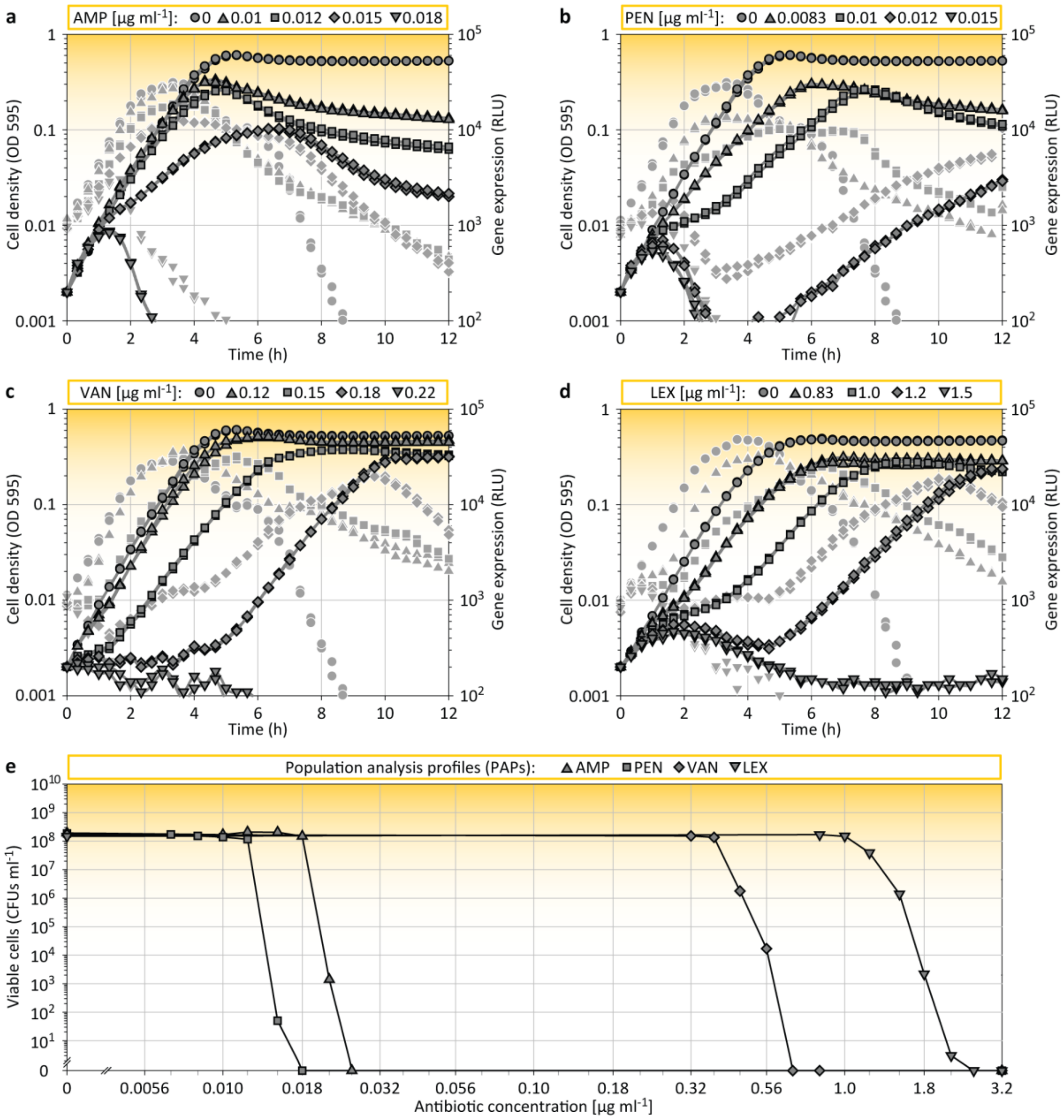

Supplementary Figure 5 | Growth-inhibition profiles and population analysis profiles of bacteriolytic drug treatment. a-**d**, Plate reader assay sets in duplicates measuring cell density (closed symbols) and gene expression (symbols without outline) of *S. pneumoniae* D-PEP22 growing in the presence of concentration series of the bacteriolytic antibiotics ampicillin (AMP) (**a**), penicillin G (PEN) (**b**), vancomycin (VAN) (**c**), and cephalexin (LEX) (**d**). **e**, population analysis profiles of bacteriolytic drug treatment by plating pre-cultured D-PEP22 cells (OD 0.1) in the presence of concentration series of bacteriolytic antibiotics and counting colony forming units (CFUs ml^{-1}) after overnight incubation; average values of duplicates are shown. Minor deviations in antibiotic susceptibility between cells of liquid culture assays and plating experiments can be observed when comparing the same annotated drug concentration; this could be either explained by differences in pharmacokinetics, or by a partial inactivation of the bacteriolytic drugs upon contact with the 55°C liquid Columbia agar during plating.

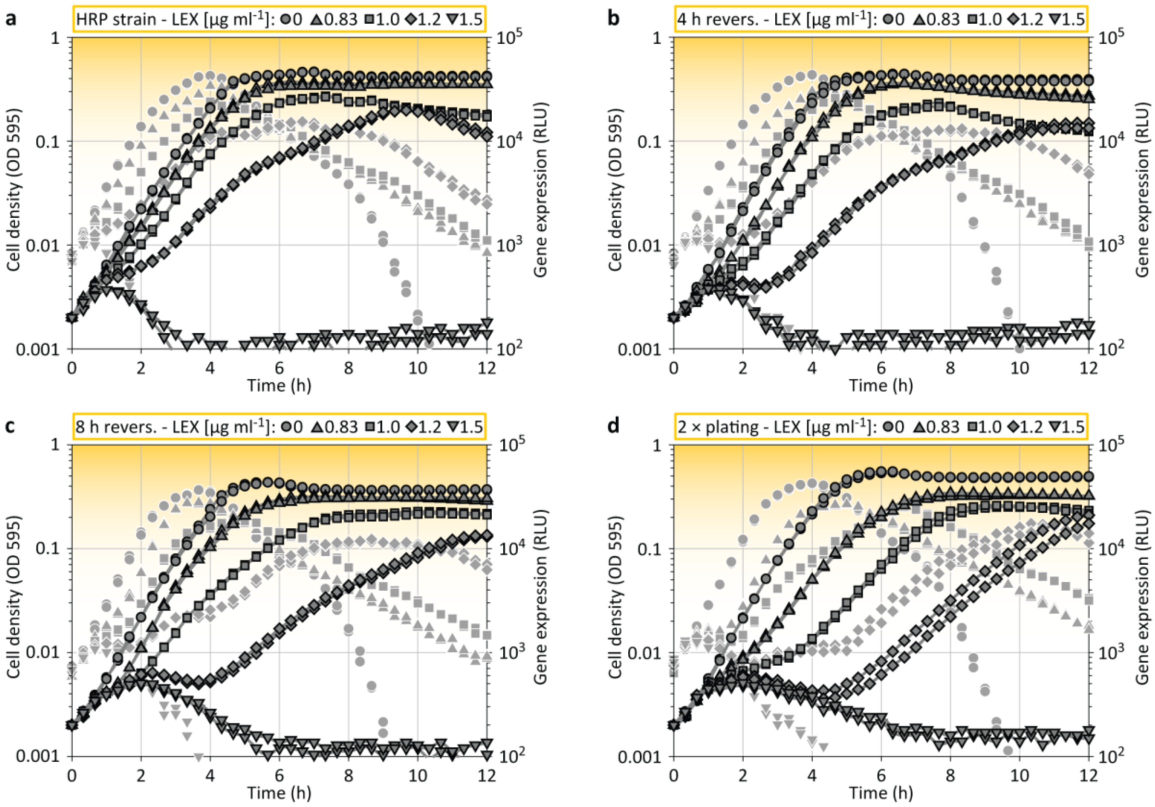

Supplementary Figure 6 | Heteroresistance phenotype (HRP) reversion assay. a-**d**, Plate reader assay sets in duplicates measuring cell density (closed symbols) and gene expression (symbols without outline) of a *S. pneumoniae* D-PEP22 HRP population (strain 1; isolated from a plate containing 1.5 µg ml^{-1} cephalexin) growing in the presence of a concentration series of cephalexin (LEX). Cells were tested for antibiotic susceptibility directly after pre-cultivation in the presence of cephalexin (**a**), after additional cultivation in antibiotic-free medium for 4 hours (**b**), 8 hours (**c**), and after re-plating twice without antibiotics (**d**).

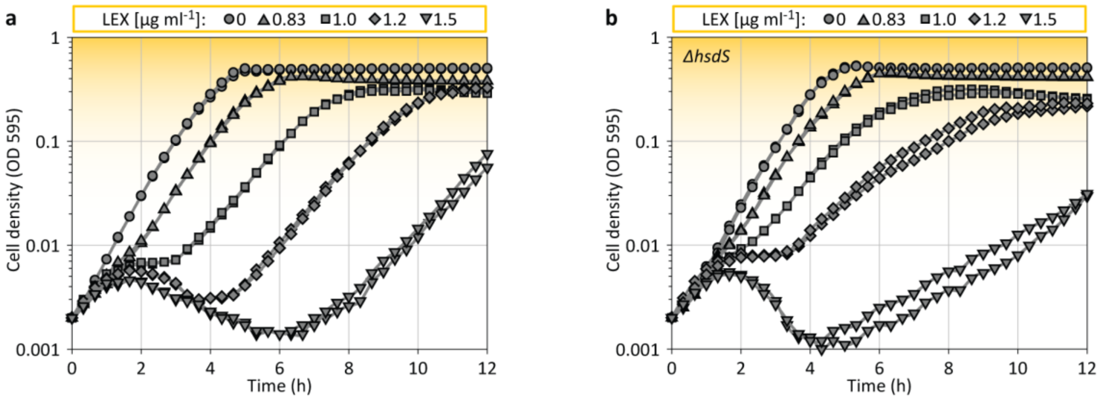

Supplementary Figure 7 | Heteroresistance phenotype (HRP) development of *S. pneumoniae* D39 *ΔhsdS*. a-**b**, Plate reader assay sets in duplicates measuring cell density of *S. pneumoniae* D-PEP22 (**a**) and D39 *ΔhsdS* (**b**) growing in the presence of a concentration series of cephalexin (LEX). Note that minor (day-to-day dependent) concentration deviations of cephalexin in growth medium master mixes strongly influence the time point of emergence of HRP populations. For instance, in this set of experiments the HRP in wild type cells also appears at 1.5 µg ml^{-1} cephalexin while this is not the case in the set of experiments shown in Figure 3b.

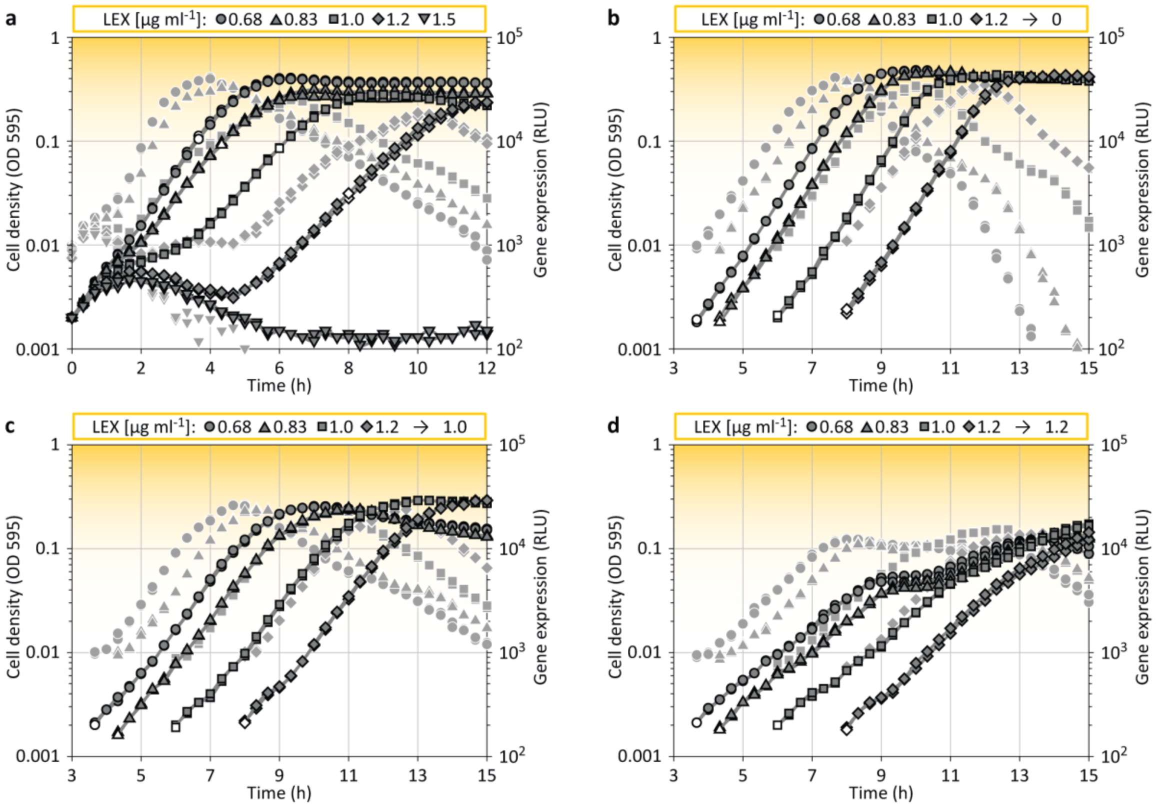

Supplementary Figure 8 | Comparison of HRP populations originating from different concentrations of cephalexin. **a**-**d**, Plate reader assay sets in duplicates measuring cell density (closed symbols) and gene expression (symbols without outline) of *S. pneumoniae* D-PEP22 growing in the presence of cephalexin (LEX). D-PEP22 populations arising from treatment with 0.68, 0.83, 1.0, and 1.2 µg ml^{-1} cephalexin (**a**), upon reaching OD 0.1 (closed white symbols; OD 0.03 in case of the 1.2 µg ml^{-1} cephalexin treatment), were re-diluted and transferred to 0 µg ml^{-1} (**b**), 1.0 µg ml^{-1} (**c**), and 1.2 µg ml^{-1} (**d**) cephalexin.

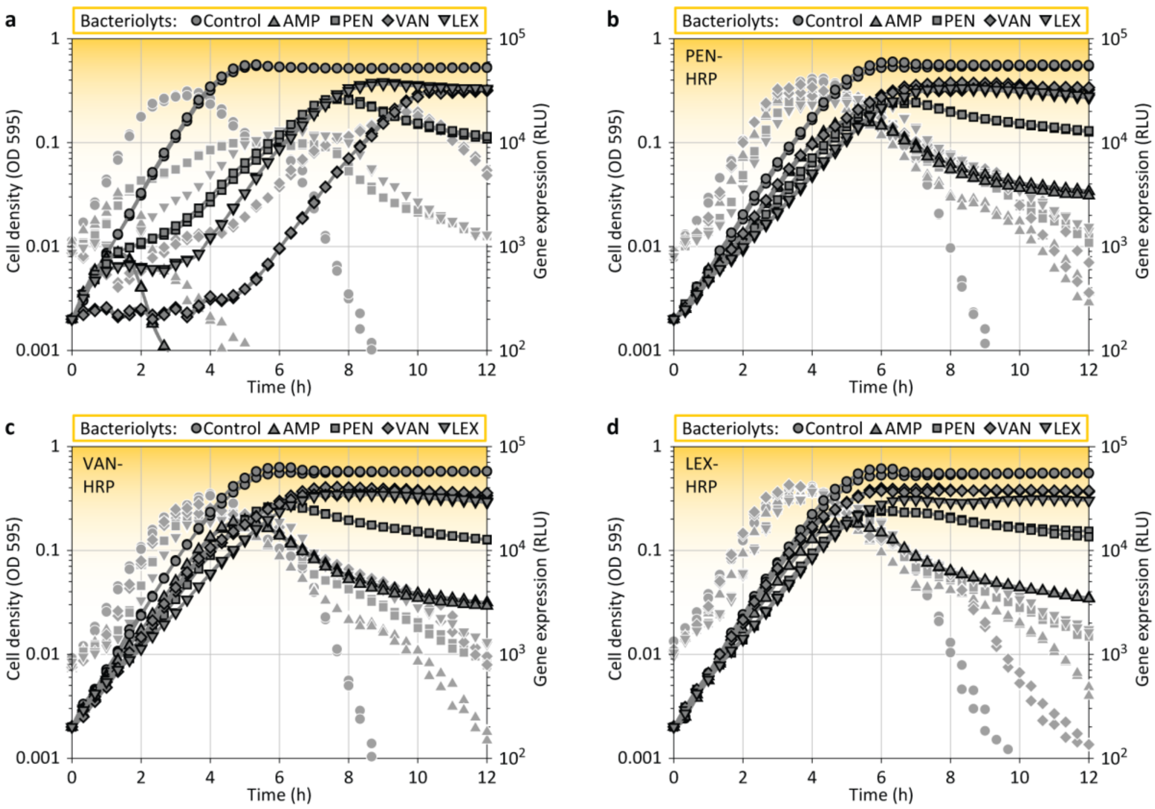

Supplementary Figure 9 | Comparison of HRP populations originating from different bacteriolytic drugs. a-**d**, Plate reader assay sets in duplicates measuring cell density (closed symbols) and gene expression (symbols without outline) of *S. pneumoniae* D-PEP22 growing without antibiotics (Control), in the presence of 0.018 µg ml^{-1} ampicillin (AMP), 0.01 µg ml^{-1} penicillin G (PEN), 0.18 µg ml^{-1} vancomycin (VAN), and 1.0 µg ml^{-1} cephalexin (LEX). Comparison of a wild-type D-PEP22 population (**a**) to HRP isolates originating from treatment with penicillin G (**b**), vancomycin (**c**), and cephalexin (**d**).

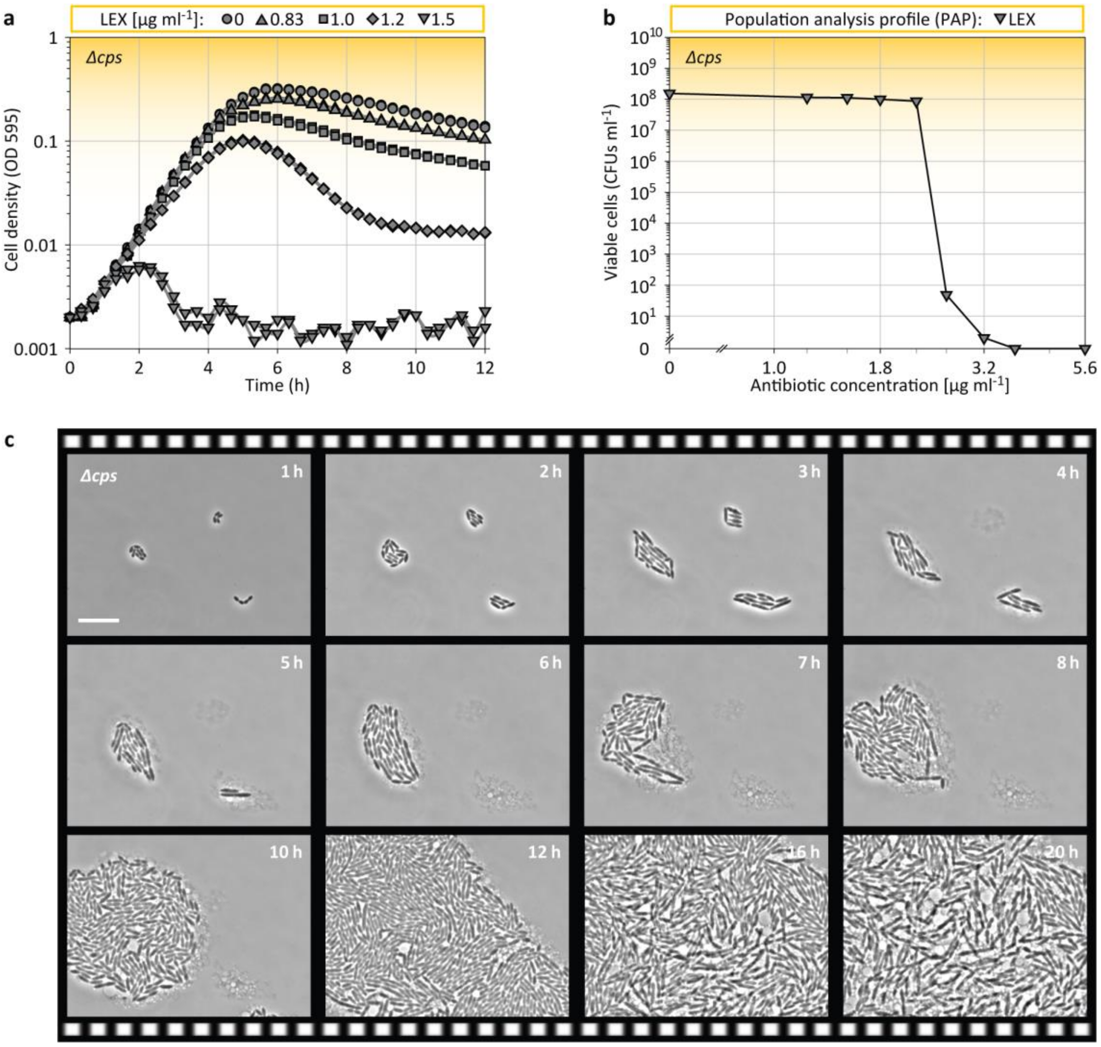

Supplementary Figure 10 | Growth-inhibition profile and population analysis profile of cephalexin-treated *S. pneumoniae* D39 *Δcps*. **a**, Plate reader assay sets in duplicates measuring cell density of *S. pneumoniae* D39 *Δcps* growing in the presence of a concentration series of cephalexin (LEX). **b**, Population analysis profile of cephalexin treatment by plating pre-cultured D39 *Δcps* cells (OD 0.1) in the presence of a concentration series of cephalexin and counting colony forming units (CFUs ml^{-1}) after overnight incubation; average values of duplicates are shown. **c**, Still images (phase-contrast microscopy) of a time-lapse experiment of D39 *Δcps* cells growing on a semi-solid surface containing 1.0 µg ml^{-1} cephalexin. Scale bar is 10 µm.

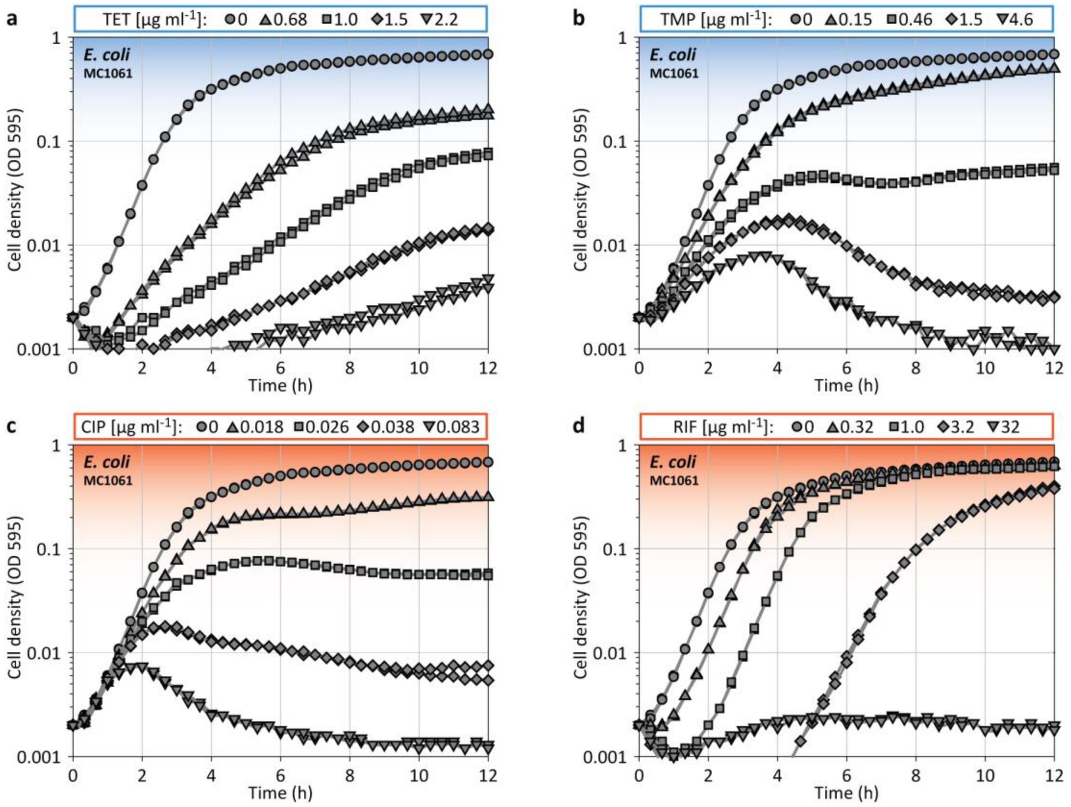

Supplementary Figure 11 | Growth-inhibition profiles of *E. coli* MC1061. a-**d**, Plate reader assay sets in duplicates measuring cell density of *E. coli* MC1061 growing in the presence of concentration series of tetracycline (TET) (**a**), trimethoprim (TMP) (**b**), ciprofloxacin (CIP) (**c**), and rifampicin (RIF) (**d**). Note that trimethoprim is classified bacteriostatic in *E. coli* (in contrast to bactericidal in *S. pneumoniae*), and growth of MC1061 cultures is affected more promptly in comparison to *S. pneumoniae* D39 cultures, in line with inhibition profiles of bacteriostatic drugs in the pneumococcus; the direct impact might be mediated via the SOS response that arrests cells upon detecting DNA damage. Rifampicin growth-inhibition profiles suggest the emergence of subpopulations displaying decreased susceptibility.

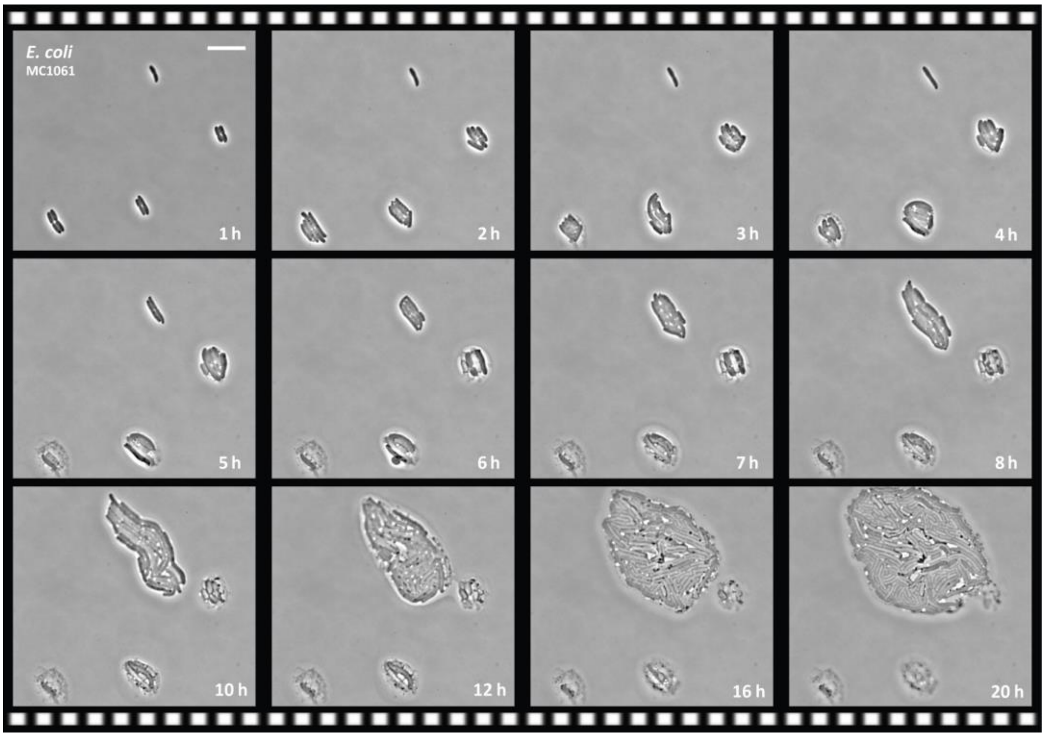

Supplementary Figure 12 | Time-lapse microscopy of *E. coli* MC1061 treated with rifampicin. Still images (phase-contrast microscopy) of a time-lapse experiment of MC1061 cells growing on a 10% polyacrylamide slide that was incubated in LB medium containing 10 µg ml^{-1} rifampicin; variations in growth and survival between recorded cells confirm the emergence of subpopulations with decreased susceptibility towards rifampicin. Note that cells from an isolated single colony were used. Scale bar is 10 µm.
